# The heterogeneity of aqueous solutions: the current situation in the context of experiment and theory

**DOI:** 10.3389/fchem.2024.1456533

**Published:** 2024-09-26

**Authors:** German O. Stepanov, Nikita V. Penkov, Natalia N. Rodionova, Anastasia O. Petrova, Angelina E. Kozachenko, Alexander L. Kovalchuk, Sergey A. Tarasov, Vsevolod A. Tverdislov, Alexander V. Uvarov

**Affiliations:** ^1^ Department of General and Medical biophysics, Medical Biological Faculty, N.I. Pirogov Russian National Research Medical University, Moscow, Russia; ^2^ Research and Development Department, OOO "NPF "Materia Medica Holding", Moscow, Russia; ^3^ Institute of Cell Biophysics RAS, Federal Research Center “Pushchino Scientific Center for Biological Research of the Russian Academy of Sciences”, Pushchino, Russia; ^4^ Department of Biophysics Faculty of Physics, Lomonosov Moscow State University, Moscow, Russia; ^5^ Department of Molecular Processes and Extreme States of Matter, Faculty of Physics, Lomonosov Moscow State University, Moscow, Russia

**Keywords:** water model, water cluster, supramolecular structures, H-bonds, water heterogeneities

## Abstract

The advancement of experimental methods has provided new information about the structure and structural fluctuations of water. Despite the appearance of numerous models, which aim to describe a wide range of thermodynamic and electrical characteristics of water, there is a deficit in systemic understanding of structuring in aqueous solutions. A particular challenge is the fact that even pure water is a heterogeneous, multicomponent system composed of molecular and supramolecular structures. The possibility of the existence of such structures and their nature are of fundamental importance for various fields of science. However, great difficulties arise in modeling relatively large supramolecular structures (e.g. extended hydration shells), where the bonds between molecules are characterized by low energy. Generally, such structures may be non-equilibrium but relatively long-lived. Evidently, the short times of water microstructure exchanges do not mean short lifetimes of macrostructures, just as the instability of individual parts does not mean the instability of the entire structure. To explain this paradox, we review the data from experimental and theoretical research. Today, only some of the experimental results on the lifetime of water structures have been confirmed by modeling, so there is not a complete theoretical picture of the structure of water yet. We propose a new hierarchical water macrostructure model to resolve the issue of the stability of water structures. In this model, the structure of water is presented as consisting of many hierarchically related levels (the stratification model). The stratification mechanism is associated with symmetry breaking at the formation of the next level, even with minimal changes in the properties of the previous level. Such a hierarchical relationship can determine the unique physico-chemical properties of water systems and, in the future, provide a complete description of them.

## Graphical Abstract

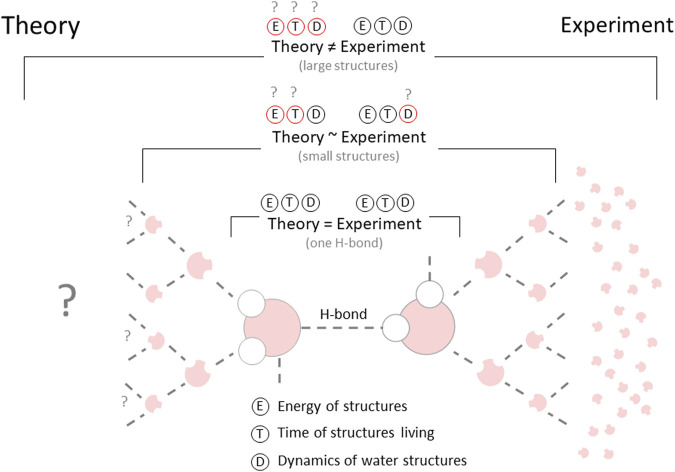

## 1 Introduction

Since it is impossible to overestimate the importance of water for humanity and the biosphere, there is no doubt about the tremendous significance of research on its physico-chemical properties. This is why this area currently attracts considerable attention and has become the focus of many theoretical and experimental studies.

Despite water molecules being simple, creating an accurate model of water proved difficult. No current model can fully and unambiguously describe their interactions in the liquid state based on a wide range of physical and chemical properties. On the one hand, there have been attempts to describe water by standard methods used in the theory of fluids. For example, the analysis of basic pair correlation functions, which is standard in the physics of fluids, for H-H, O-H, and O-O pairs from neutron scattering data as well as from modeling results (Soper et al., 1997), makes it possible to trace correlations up to distances of the order of angstroms, which, of course, is not sufficient for a network of hydrogen bonds.

Today, research in the field of water modeling is actively developing and has many achievements, but describing even small groups of water molecules with good accuracy is a problem that has yet to be solved. The point is that the standard approaches used in the physics of fluids (analysis of the simplest pair interaction potential and investigation of the short-range order using the pair distribution function) were initially applied to water and aqueous solutions just as to any liquid, and a similar approach is used in current literature. Usually, model calculations for liquids require considering many-particle interactions. However, in water, there is an additional complex network of hydrogen bonds with short lifetimes and polarization of molecules relative to each other, which greatly complicates the task of theoretical analysis and calculation of the interaction potential between water molecules. Thus, one of the main problems in water modeling is the correct description of the network of hydrogen bonds.

The unique properties of water are also well known and have been documented experimentally (Eisenberg and Kauzmann, 2005). They include, firstly, an anomalously high heat capacity and temperature of vaporization compared to other hydrates in this group. Another property that interferes with the modeling of aqueous systems and represents the most striking manifestation of bond rearrangement is the anomalous relationship between density and temperature, with a maximum at approximately 4°C for H_2_O and 11°C for D_2_O. Strange as it may seem, most of the potentials used in molecular dynamics (MD) do not describe this feature accurately enough, and an artificial change in the potential with temperature has to be introduced for a more precise description. A disadvantage of this method is that it lacks any physical justification and is introduced just for the fitting of the experimental data. In this case, accurate modeling using the potential cannot be achieved.

The anomalous properties of water also include nonmonotonic temperature dependencies of heat capacity (with a minimum at 35°C), isothermal and adiabatic compressibility (with minimums at 46°C and 60°C, respectively), and a very high experimental surface tension constant compared to theoretical predictions and models (Cisneros et al., 2016).

Increasing attention has also been paid to the anomalous temperature and concentration dependence of diffusivity (Andersson, 2023; Helm and Merbach, 1999). Diffusion in liquids is determined by the transitions (jumps) of molecules and their mutual exchange, and if this process is not described correctly, then no accurate modeling is possible.

Anomalous properties of water are thought to be associated with the presence of hydrogen bonds (Andersson, 2023) and the occurrence of heterophase systems (structures), which reorganize with changes in temperature and pressure. For many years, this viewpoint dominated the development of theoretical models of water properties. However, in recent decades, due to the development of novel experimental methods and the continued search for adequate theoretical models to describe experimental results, the emphasis has shifted significantly.

New experimental data indicate the rapid exchange processes occurring in water (Helm and Merbach, 1999; Zhang et al., 2022). The time of these exchange processes and the lifetime of water systems (structures) are considered to be commensurate and similar in magnitude. However, exchange processes do not necessarily lead to the destruction of structures, and therefore the issue of the relationship between the rate of exchange processes and the lifetime of structures is currently the subject of active discussion.

Models of hydrogen bond jumping incorrectly describe changes in the position of molecules in time and space, despite plausibly describing diffusion, which should be related to the structure of the solution. As I. Marcus stated in (Marcus, 2009) “Although it is generally accepted that water is a highly structured liquid, there is little agreement on how to express the structure quantitatively and how to measure it experimentally.” However, an analysis of the diffusion of polyatomic ions makes it possible to consider both the mechanism of ion rotation and the influence of water molecules on this process (Banerjee and Bagchi, 2019).

With time, significant changes in experimental approaches to the study of the structuredness of aqueous solutions have occurred. In the first half of the 20th century, there were a considerable number of theories of water involving the hypothesis of the existence of long-lived structures. Roentgen was the author of one of the first two-structure models involving strong solid-state structures (Röntgen, 1892). He proposed that liquid water consists of two types of molecules with different dependencies of their properties on temperature and pressure. This model made it possible to describe the anomalies known at that time, but substantiating the separate existence of two compartments obviously required additional arguments. Other models should be mentioned, such as I.S. Fisher’s (Fisher, 1948), L. Pauling’s (Pauling, 1959), and other “exotic” water structure models (Chaplin, 2000). Attempts to describe the unique properties of water have been made in more recent research (Soper, 2019).

Today, the so-called standard Bernal model is one of the most accepted and commonly used (Bernal and Fowler, 1933). It is based on two important assertions. Firstly, if water were a simple liquid with corresponding van der Waals radii for the constituent atoms, then its theoretical density would be about 1.8 g/cm^3^ (Bernal and Fowler, 1933). However, the actual water density is close to 1.0 g/cm^3^. From this, it follows that the water must be loose. Secondly, it is known that the binding energy between water molecules is very high compared to other liquids, which means that water is dense. These two conclusions are opposite, and this is where the idea of the structure of water depending on the network of hydrogen bonds comes from (Lokotosh et al., 2003). It defines water as a heterogeneous medium that contains complex structures (clusters). This idea was confirmed by the recent work by Naserifar and Goddard III (Naserifar and Goddard, 2019). Utilizing the RexPoN force field, which is based entirely on quantum mechanics without empirical data, they revealed that water can be described as a polydisperse branched polymer where charge and polarization change dynamically. The study demonstrated that each water molecule, on average, forms approximately two SHBs at 298 K. This is two times less than in ice, which means the possibility of the formation of polymer-like chains with varying lengths and branching points. However, the lifetime of these structures is still a subject of discussion.

The key point is that, in reality, we never deal with absolutely pure water, and it would be logical to assume that impurities/ions contained in the aqueous solution can be the source of the formation of these clusters. In contrast to hydronium, impurity ions are hydrated, meaning that they are centers of structure formation due to the appearance of non-vanishing charges. It is well known from the theory of solutions that a decrease in concentration leads to an increase in the Debye radius and a decrease in the influence of other ions (Pitzer, 2018). This key rectification makes ions the most important element of real (in contrast to ideal) water. The effect of large amphiphilic molecules on water differs significantly from that of small ions. However, the consequence of such an effect is also obvious: these molecules are the centers of the formation of water structures both within the volume and on the surface of the aqueous solution. Problems also arise when describing such structures on the surface. The theoretical analysis is based both on the study of Gibbs adsorption isotherm (non-ionic models) and on models involving the analysis of ionic interactions (Prosser and Franses, 2001). Apart from that, the study of surface properties and the interaction between the volume and surface of water is a separate subject. We can only state that it is apparently even more complicated than the studies of bulk water properties. Even at very low concentrations of impurities, a complex surface structure appears, with several phase transitions in the near-surface layer (Adamson, 1976).

Thus, there are two directions of research into the structuring of water: experimental and theoretical. Both of these approaches provide complementary, but not complete, conclusions. Theoretical calculations are actively developing and provide many excellent conclusions, but they still cannot describe the heterogeneity of water systems with sufficient accuracy. At the same time, experimental approaches can estimate the lifetime of hydrogen bonds but are not accurate enough in describing the dynamics of water structures.

In this review, we discuss these topics so that the reader has an adequate understanding of the complexity of the problem. We will consider the range of water properties for which there are serious discrepancies in the estimates obtained with existing models versus experimental results. However, water only at ambient temperature and above will be considered. The structure of water at low temperatures is currently a distinct and equally significant area of research in the general theory of water, but will not be discussed here. This review will also not focus on the water bound on substrates, in pores, in nanotubes, etc., when the interaction with the surface has a decisive effect on the formation of the water structure. Finally, we will propose a model of the symmetry of water systems as a physico-chemical mechanism for stabilizing the heterogeneity of water.

## 2 Computer modeling and the possibilities of predicting the structure of water

The opinion on the short lifetime of supramolecular structures of water that has become common today is based solely on the results of model representations instead of direct experimental results. *A priori*, it was assumed that a certain theoretical model of water exhaustively described all its thermophysical properties, since it would be otherwise impossible to draw a correct conclusion about other properties of water. If a model does not adequately describe all thermophysical properties, then its application involves a high probability of erroneous conclusions. For example, based on experimental results, it becomes obvious that structurally liquid water is not an intermediate state between steam and ice but a system that is much closer to the solid state (and, therefore, a structured system), which has a non-linear energy distribution. The energy required for the complete evaporation of liquid water is 2,260 kJ/kg, while the energy required for the ice-water phase transition is only 334 kJ/kg.

In the theoretical description (modeling) of water properties, the choice of molecular models of diffusion plays a particularly important role. However, averaged Lennard-Jones potentials cannot be used to describe the structure of water because of their association with a fixed geometry of point charges or the possibility of modeling only small clusters. TIPnP and SPC models that apply effective pairwise force fields during calculations are now being considered less and less often, but more sophisticated models such as AMOEBA (Medders et al., 2013; Yu et al., 2013; Yu et al., 2003), TTM (Burnham and Xantheas, 2002a; Xantheas et al., 2002; Burnham and Xantheas, 2002b; Burnham and Xantheas, 2002c; Fanourgakis and Xantheas, 2006), DDP2 (Kumar et al., 2010), POLIR (Mankoo and Keyes, 2008), POLI2VS (Hasegawa and Tanimura, 2011), SWM (Caldwell and Kollman, 1995; Chen et al., 2000), COS (Yu et al., 2003; Kunz and van Gunsteren, 2009; Kiss and Baranyai, 2013), BK3 (Sokhan et al., 2015), ASP-W (Paesani et al., 2010; Paesani et al., 2009), SCME (Wikfeldt et al., 2013), and others do not provide a full description of the thermodynamic properties of water in the liquid state and during the transition from the liquid to the gas state. To use one or another model, it is necessary to check its compliance with the parameters required for the calculation (Cisneros et al., 2016; Brini et al., 2017; Wang et al., 2013; Buck et al., 2014; Bushuev, 1997). Despite the impressive list of models and their complexity, none can be used to describe long-lived structures or to state their absence because, in order to simplify calculations, they do not account for the long-range interactions of molecules, which play a very significant part.

### 2.1 Existing approaches for water modeling

Water modeling has undergone several stages. Relatively simple models were initially developed in attempts to explain the diversity of water properties using rather obvious considerations, for example, separating water into several phases with different properties to set a simple interaction potential between molecules depending only on the position of the nuclei, etc. At present, such hypotheses are no longer of great interest, primarily because they have been put forward for a long time but have not brought any breakthroughs in the theory of the structure of water or the theory of liquids in general. However, relatively simple models have recently emerged, claiming accuracy similar to that of complex models but only for some considered properties.

As mentioned in the introduction, any model must describe the basic anomalous properties of water to provide a good agreement between the experiment and the theory. At the subsequent stages of the modeling, with an increase in computing power, the idea of a simple description of a liquid as the interaction of a certain number of molecules with a given potential appeared, harrowing the emergence of the method based on MD. Originally, this method was intended to replace numerous empirical thermodynamic characteristics with certain parameters of molecule interaction potential. The computing of the forces acting on the molecules, explains the understanding of their trajectories and interactions. These calculations take into account the impact of both deterministic and stochastic forces on the particle’s motion, providing a comprehensive model of the dynamics. Unfortunately, this approach is difficult to apply to water at present due to the complexity of the problem. If the potential is adjusted based on the target characteristic (for example, to accurately describe the dependence of density on temperature), then such modeling loses its physical meaning if the remaining properties of water are not analyzed. Moreover, classical MD models often rely on empirical potentials, such as Lennard-Jones and Coulomb, to describe interactions between water molecules, which may not accurately capture long-range interactions, typical for water clusters, and the polarization effects that are significant in water. Due to the current lack of a unified theory, there are numerous empirical models for describing water. In this case, the methods are well known as they focus on thermophysical and electrical parameters, and this testing is currently receiving considerable attention.

A significant breakthrough in understanding the physico-chemical properties of water was achieved by modeling its structural heterogeneities. This is how the dynamics of the behavior of complex systems is studied, with the identification of structures (Lynden-Bell et al., 2001). Empirical force fields are used to describe such complex systems. The implementation of empirical force fields is based primarily on experimental results rather than theoretical prediction (Pascal et al., 2012). Using this approach, spatial and temporal heterogeneities were studied. For example, to study the heterogeneity of the condensed phase, it was possible to simulate water in the core-halo structure. It is thanks to the study of particle relaxation between these two compartments (core and surface) that it is possible to assess how water structures are formed and stabilized (Konishi et al., 2015). Other examples of the use of empirical force fields in the study of water include estimating the temperature dependence of the surface or bulk state of water, approximating freezing point calculations, and calculating the lifetime of hydrogen bonds. Water modeling confirmed that the hydrogen bond lifetime is of the picosecond order, indicating the dynamic nature of aqueous solutions (Burnham et al., 2006; Smith et al., 2005; Rawat and Biswas, 2014). This dynamic nature reveals features of their interaction with ions that are difficult to demonstrate experimentally. Thus, the hydration shell around the anions is not rigid, as previously thought. Instead, it exhibits lability, which means that water molecules can quickly switch between different hydrogen bonds, which affects their orientation and dynamics (Brett and Rohani, 2020). Investigating the heterogeneities using the HIPPO potential, based on electron density modeling, also helps to understand the structural heterogeneity of water (Rackers et al., 2021).

At present, attempts to make the models of pairwise interactions more complex by including additional hydrogen bonds are also being made within the scope of the simplest models of the TIP4P type, modernized to implement such techniques (Henchman, 2016), but even the number of hydrogen bonds per molecule remains a subject of discussion (Muthachikavil et al., 2021). Moreover, using *ab initio* MD simulations hydrogen bonding and charge transfer between donor/acceptor water molecules can be obtained. A relatively simple model based on the energy flow through donor-acceptor pairs of water, allows quantifying the degree of reorganization of the local network of hydrogen bonds in the water structure. When using the second-generation Car-Parrinello MD, the parameters of the calculated system, consisting of 64 water molecules in a liquid volume with a length of 12.43 Å, fully corresponded to the parameters of liquid water under normal conditions. The accuracy of this method is quite high because it is not necessary to consider the entire complexity of hydrogen bonds, which simplifies calculations and reduces the likelihood of error (Bertie and Lan, 1996; Ojha et al., 2018a). The adaptation of these theoretical models to aqueous solutions in the presence of large-molecule impurities, such as biopolymers, is much more acceptable (Hatakeyama and Hatakeyama, 2017). In this case, water is divided into several types that differ in their properties. However, in this case, the association with a polymer logically provides for such a separation. A similar situation arises in the analysis of liquid near electrodes (Kanemaru et al., 2017). The same reasoning can be applied to water in biological structures, nanosized tubes, etc.

Another method, path integral molecular dynamics (PIMD), provides a convenient way to efficiently incorporate nuclear quantum effects (NQE) into atomistic simulations. NQE have the dual nature in hydrogen-bonded systems including water, what can be explained by the idea of “competing quantum effects” (Green et al., 1986; Ceriotti et al., 2016). This concept posits that quantum effects can both strengthen and weaken hydrogen bonds depending on the specific vibrational modes involved. For instance, nuclear effects can lead to an extension of the O–H covalent bond, making protons more delocalized between water molecules and potentially strengthening the hydrogen bond. Although the nature of NQE in chemical processes involving hydrogen atoms, solvated protons, hydroxide ions, and water itself is fairly well known, the extent and magnitude of the effects on various static and dynamic properties remain controversial. Using PIMD simulations, it has been shown that proton delocalization and tunneling are more pronounced in water under high pressure or low temperature, contributing to the unique phase behavior of ice and supercooled water. Instantaneous fluctuations of the ground-state frequencies are calculated using the wavelet method of time series analysis. In particular, the dynamics of frequency oscillations are determined using FTCF (frequency time-correlation function) and the three-pulse photon echo (S3PE) slope. In addition, the lifetime and reorientation scales of hydrogen bonds, as well as the time-dependent frequency probability distribution, three-point frequency correlation, and frequency structure correlation functions are calculated (Hare and Sorensen, 1992; Ojha et al., 2018b).

In what cases do these theories currently refer to bulk pure water? Primarily, there are attempts to separate a continuous distribution by angles, sizes, and types of hydrogen bonds into two classes. Such mixture models are often used to analyze water near the freezing point and supercooled water (Moore and Molinero, 2009). A detailed analysis of this approach is presented in (Soper, 2011) where water is represented as a mixture of two parts that interpenetrate each other, but form hydrogen bonds only with the molecules of the other (not its own) part. An example of the division into two liquids under normal conditions, for ordinary and two types of water, from the point of view of different times of orientation of molecules: fast with “weak” hydrogen bonds, and slow with “strong” ones (Woutersen et al., 1997).

As mentioned above, the most important feature of water is the combination of the relative simplicity of one molecule with the complexity of mutual interaction between molecules (Kaatze, 2018). It is obvious that, as in the theory of simple liquids, a transition from the model of pair interactions to the model of many-particle interactions is necessary. Such models were developed in the second half of the 20th century and were thought to be sufficient to describe all the properties of water.

First should be mentioned the so-called “effective pairwise force fields,” or “rigid non-polarizable” models, such as TIPnP (and especially TIP4P) and SPC (Horn et al., 2004; Berendsen et al., 1987). The main feature of such models is the specification of the potential as a combination of the Coulomb forces and the Lennard-Jones potential. For four decades, these models have often been used to analyze structures due to their relative simplicity, as they replace real interactions with a set of certain effective forces. Various effects associated with long-lived structures were also considered based on these models.

Researchers have revisited these models to improve them because their accuracy in reality is not inferior to the accuracy of more complex models that consider polarization at significantly lower computational costs. According to current trends, making them more complex is not required because it does not lead to significant improvements. Obviously, this situation does not allow us to consider using such models to describe the evolution of supramolecular structures in water.

For example, the TIP4P/2005 model reliably predicts the surface tension coefficient, density, and self-diffusion coefficient (Muthachikavil et al., 2021). This model makes it possible to choose parameters for an adequate description of the phase diagram, but is not suitable for an accurate description of compressibility and freezing temperature. The TIP5P model was specially adjusted to accurately describe the temperature dependence of density, and much attention is currently being paid to its further development (Mahoney and Jorgensen, 2000). The robust three-body water simulation model (E3B) has several advantages over the TIP4P model (Tainter et al., 2011). An analysis of activation energies and transitions in rigid nonpolarization models can be found in (Piskulich and Thompson, 2021), but in general, preference is given to the TIP4P/2005 and E3B3 models.

### 2.2 Accounting for polarization

The main drawback of simple models of many-particle interactions in their classical form is obviously the fact that they do not account for electronic displacements. If polarization is not factored in, the interaction potential depends only on the position of the nuclei. Actually, this is a major problem for the entire theory of liquids: many-particle interaction automatically leads to different polarizations. Therefore, the standard theory of electrostatics does not operate, considering the additivity of charged particle interactions. For water, the situation becomes more complicated due to the presence of hydrogen bonds, which significantly increase the interaction energy compared to conventional van der Waals forces and shift the position of electrons. Today, the research is primarily focused on many-particle interaction potentials with polarization taken into account.

The computer modeling of water was first undertaken in 1969 (Barker and Watts, 1969), and *ab initio* simulation was performed in 1993 (Laasonen et al., 1993). Thus, modeling has a long history. Since then, no universal model has been created, and the number of proposed options with various types of simplifications is still growing. Under these conditions, some researchers naturally started using the generalized approach, in which any model is tested for all the basic water properties. This logic seems very promising. With the development of digital methods, the applicability of simple models has been questioned. In the last decade, reasoned calculations and comparisons with complex models have appeared, showing the low accuracy of the models that were used in the early stages of simulation development.

A detailed analysis of the models themselves used today in numeric calculations is not the goal of this section, especially as such a description would be very long. Importantly, macrostructures in water are determined by many-particle interactions, and their influence on the thermodynamic parameters of water is minor. Therefore, models that cannot accurately describe even the main thermodynamic characteristics of water would be unable to adequately describe large-scale structures.

The limitations imposed on these models have been studied in detail for a long time. Apparently, the publication (Guillot, 2002), which has more than 1,100 references, raised the issue of the applicability of models and the empirical potentials used in them quite extensively for the first time, discussing more than 50 models and the issues of transferability of results, allowance for quantum mechanical effects, etc. The paper (Vega and Abascal, 2011), which appeared much later, retained only the most frequently used simple models, and a fairly wide list of verification tests was developed. These include vaporization heat, melting heat, parameters at the critical point, surface tension coefficient, temperature dependence of density, isothermal compressibility coefficient, static dielectric constant, properties in the gas phase, dependence of heat capacity on temperature, equation of state at high pressures, self-diffusion coefficients, shear viscosity coefficient, phase diagram, and parameters of the radial distribution function determined experimentally. Five models were considered (TIP3P, TIP5P, TIP4P, SPC/E, TIP4P/2005) and rated. TIP3P was rated the lowest and TIP4P/2005 the highest. Nevertheless, it was noted that none of the presented models described all the properties with sufficient accuracy. Another method for comparison requires using sophisticated models and quantum mechanical calculations that make it possible to estimate the accuracy of simpler models.

In the context of the importance of more accurate calculations, at least two components of intermolecular forces should be singled out: the average force, which can be described by the interaction potential and standard methods of molecular classical theory (Vega and Abascal, 2011); and bonds, which can be described by the Schrödinger equation and electron density clouds (Wang et al., 2004; Willow et al., 2015).

This approach is considered fundamental and is designated (often groundlessly) as *ab initio*, although the methods for solving the Schrödinger equation in quantum chemistry are known to be approximate. The task of determining the electron cloud for given coordinates of the nuclei is ambiguous even in the Born-Oppenheimer approximation. The interaction potential in different approximations is determined by two-particle, three-particle, and many-particle interactions. Many of these conditions are contained in the MB-Pol model, which takes account of many-particle interactions, and is a combination of classical and quantum mechanical approaches. Moreover, this model quantitatively describes various properties of water, including the vibration–rotation tunneling spectrum of the water dimer, isomeric equilibria, vibrational spectra of small water clusters, and the structural, thermodynamic, and dynamical properties of liquid water across different phases (Lambros and Paesani, 2020). Also, similarly to MB-Pol, the TTM3-F and TTM4-F models have been developed to improve the description of vibrational spectra and polarizability surfaces of water, using empirical representation of charges and simulations of water molecule’s polarizability.

An analysis of small-size clusters (Willow et al., 2016; Góra et al., 2011) shows that, for example, three-particle interactions provide 15 to 20 percent of the energy, while the contribution of four-particle interactions is approximately 1 percent. The change in the energy of formation of structures may be small. Small energies of large structures are difficult to model with approximate series expansion. This approach can be a source of inaccuracy in heterogeneity modeling.

In recent years, calculations involving large clusters of water have also been published, which allow comparing the contribution of individual components of the applied models. In the study, different accuracy of different models was shown (from less accurate q-TIP4P/f or E3B to much more accurate MP2 and HBB2-pol). The authors pay special attention to the prospects of the HBB2-pol model for studying condensed phases (Medders et al., 2013).

**FIGURE 1:**
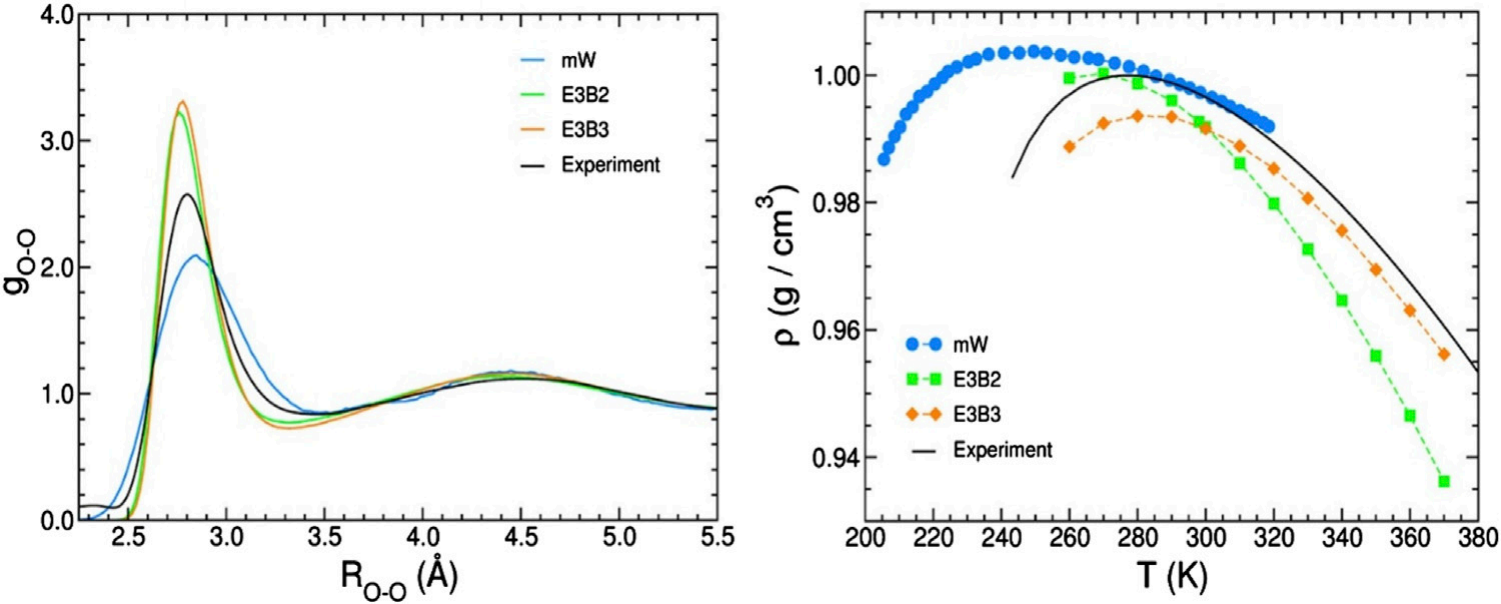
Comparison of experimental data obtained for liquid water and simulations using several commonly used models for pair correlation function O-O (left) and temperature dependence of density (right) (Cisneros et al., 2016).

However, it should be noted that most models solve only one problem, for example, calculating thermodynamic parameters or the ability to accurately describe the interaction of a group of water molecules, etc. This is an absolutely logical and correct approach, but it also forces us not to obtain a systemic understanding of water modeling under various conditions. For more systematic assessment, obviously, more complex models should be used that take into account all the features of interactions in water. A comparison of such models is presented in (Cisneros et al., 2016). Simulations using two types of models (a coarse-grained mW model and empirical E3B models) were compared to the corresponding experimental data. In particular, the authors compared the oxygen–oxygen radial distribution functions of liquid water at ambient conditions derived from X-ray diffraction measurements and the temperature dependence of the density of liquid water at ambient pressure. Figure 1 illustrates that even with the simplest simulation, there are discrepancies with the results of actual experiments. In general, when using more simple models, the results can be considered approximately similar. However, in the case of complex models (Supplementary Table S1), the simulation results differ markedly from the experimental results (Cisneros et al., 2016). Thus, only some properties of water can be described using these models, and a model describing a wide range of water properties does not yet exist.

Below, we list some popular models. The AMOEBA model (Ren and Ponder, 2003) in different variations is a classic version of MD calculation, allowing for polarization and displacement of the electron cloud. Comparison with non-polarization models demonstrates higher data accuracy when polarization is taken into account (Muthachikavil et al., 2021). SIMBA (Piquemal et al., 2003) is a method that introduces corrections for orbital overlap in order to take into account quantum effects using simple approximation, which was tested on some metal ions. TTM2 (Burnham and Xantheas, 2002a) used *ab initio* potential surface calculations for dimers, and NEMO (Engkvist et al., 1997) presented an attempt to use a simplified procedure for taking into account trimers and tetramers, applying the simplest methods of analysis of potential surfaces, etc. An example of polarization calculations from the first principles is described in (Rozsa and Galli, 2021). Incorporating polarization effects into water models enhances the accuracy of simulations by allowing for a more realistic representation of the dynamic nature of water molecules’ dipole moments. Polarization accounts for the response of water molecules to the external electric environment, which is crucial for accurately describing properties like dielectric constants, hydrogen bonding, and the behavior of water in different phases.

There are many similar comparisons in the recent literature (Wang et al., 2013; Fu et al., 2009; Zhai et al., 2023; Fu et al., 2022); they mark a radical change in the perception of such simulations. Models are currently required to pass tests for conformity with the thermal and electrical properties of water in a variety of conditions. In many cases, errors in determining the parameters may not be significant, but they are of paramount importance for identifying structures in water. All the studies primarily conclude that realistic reproduction of the structural properties of water in numerical calculations is still far from being achieved. On the other hand, in the absence of success in exact simulation, one should expect the development of simpler methods that have been used in the past and will be improved in the near future.

### 2.3 On the insufficiency of existing models

Due to the absence of a basic function for a long-range order, as is common for liquids, a radial distribution function is used to describe the characteristics of a short-range order. Some experimental methods (such as X-ray diffraction analysis) make it possible to obtain this function with some accuracy and at small distances. For water, three such functions are used (Nilsson and Pettersson, 2011): O-O, O-H, and H-H distance functions. For each of these functions, the relevant models of water structures are considered (Soper and Ricci, 2000). However, the accuracy of measuring small changes in these functions is low, and additional steps have to be taken to improve it (Fu et al., 2009). Such steps include the use of a radial distribution function, which can be determined by X-ray scattering using a diffracted beam analyzer to assess the scattering. X-ray diffraction capabilities are constantly being improved. Until recently, diffractometers allowed measurements up to 30 Å, and only recently was the upper limit expanded to 600 Å (Soper and Ricci, 2000). However, it may still be insufficient for studying structures in aqueous solutions, which may exceed these dimensions.

There is also an obvious problem with applicability of the radial distribution function to describe the structures in a liquid. This function cannot directly indicate the long-term nature of the existing structures because it is obtained by averaging over time and space and. In addition, this is just the pair correlation function that does not account for the many-particle interactions of molecules.

However, the issue of the relationship between the averaged parameters of emerging structures and the time of their existence has an important physical meaning. Thus, it is obvious that the intensive development and increasing complexity of models describing individual properties of water do not lead to the emergence of a universal model, although this improves our vision of the situation.

The development of simulation and the increase in the number of models led to the need to systematize these models and understand their potential for describing the properties of water (Guillot, 2002). At present, similar studies analyzing the full set of parameters appear regularly. Their result is disappointing, as none of the models can provide a comprehensive description of the experimentally measured physical and chemical properties of water. In such a situation, there is a return to simpler models with a large number of parameters for adjustment. The return to such models demonstrates a certain crisis in water simulation. Several such models can be cited as examples. The paper (D’Alessandro et al., 2008) optimizes the repulsive part of the Buckingham potential, which makes it possible to improve the accuracy of calculations in comparison with SPC and TIP4P models. Another example is the return to simple two-fluid models (Bernal and Fowler, 1933). Another type of approximation is provided by the introduction of different Arrhenius dependencies for jumping frequency in different temperature ranges, which makes it possible to fit the results for the coefficients of viscosity, diffusion, and dielectric relaxation time to the experimental data (Kholmanskiy, 2021).

This approach means, as always in two-fluid analysis, that the presence of hydrogen bonds changes the relationship between jumping and activation energy. Another approach is associated with an attempt to analyze the legitimacy of simplifications that are made when formulating the model and affect the result of calculations (Reddy et al., 2016).

In summary, it became clear that complicated models do not provide sufficient accuracy, and therefore there is a return to simple models, making it possible to assess the properties of water at an approximate level but with less effort. Indeed, the transition to an equilibrium state independent of the initial conditions means the presence of a universal distribution over the characteristic dimensions of the structures, which is determined by the temperature of the liquid and the concentration of impurities. Many experimental methods make it possible to study such distributions. Relaxation time is the most important parameter in this case. This problem fits within the framework of classical kinetics. The appearance and measurement of relaxation time would strengthen the argumentation of supporters of long-lived structures. By contrast, if different variants of the equilibrium size distribution arise, it is possible to assume the existence of long-term memory and study different types of water. This issue will be discussed in more detail in the next two sections.

### 2.4 The role of machine learning in improving existing models

The theory of hydrogen bonds made it possible to understand the reasons for the existence of anomalous properties of water and its low density. The development of MD in the middle of the 20th century allowed to calculate the structure of water, of course, using the pair interaction potential at that time. The development of these methods, as noted above, led to many approximations that described certain properties of water, but each property required its own approximation. Accounting for the theory of many-particle interactions, polarization, and quantum mechanical effects has made the calculations extraordinarily complicated, but at present it has not led to the required result, namely, the construction of a universal model.

Machine learning and neural networks have been used in various fields of physics and chemistry since the beginning of this century, and only recently have they attracted general attention (Behler, 2016; Heindel et al., 2023). They are also actively introduced into the calculations of interaction potentials for water, and so far, no breakthrough results in this area have been obtained. Moreover, some of the simplest approaches, for example, using the improvement of pair potentials of interactions, obviously cannot lead to a result because the parameters change when trying to include new properties in the description, a new approximation is implemented, and it worsens the description of previous properties. Meaning that training cannot improve the model.

However, modern approaches and the latest generations of neural networks may well improve the calculation results. Much attention in this regard is paid to the analysis of clusters (Abella et al., 2023; Gao et al., 2022; Herman and Xantheas, 2023; Haghighatlari et al., 2022). This analysis is much simpler than solving the complete problem, and, of course, it does not allow one to analyze large structures.

Machine learning-based modeling is actively used to study liquid water. For example, the flexible neural network potential constructed in (Cheng et al., 2019) for bulk liquid water and ice can quite accurately describe the thermodynamic and structural properties of water in accordance with experiment. The idea of using machine learning potentials (MLP) greatly expands the applicability and performance of approaches to electronic structures, making them accessible for their precise calculation of free energies and other thermodynamic properties. A recent study (Avula et al., 2023) demonstrated the potency of MLP trained on density functional theory (DFT) data, with the use of which it was possible to obtain differences in the hydration shells of Na^+^ and Cs^+^ ions, which could not be achieved with a classical non-polarizable force field. The use of MLP has allowed a more detailed understanding of the phenomenon of anomalous diffusion due to the accurate reproduction of experimental thermodynamic, structural, and transport properties of aqueous solutions.

General structure and free energy methods provide a reliable way to predict the thermodynamic properties of a variety of physical systems, such as pharmaceutical compounds, hydrogen storage materials, hydrocarbons, and metal alloys. At the same time, small errors may occur due to the choice of the fitting strategy by the neural network and the selection of finite cutoff radii used in the description of atomic media.

Another work shows that a potential trained on liquid water using machine learning can predict the density, bond energy in the crystal lattice, and vibrational properties of ice (Monserrat et al., 2020). Based on the results of this simulation, it has been shown that liquid water contains all atomic environments in different phases of ice, which provides a fundamental insight into the understanding of liquid water and ice. However, despite the popularity and successful application of MLP in thermodynamic calculations, for systems where long-range interactions are dominant and occur, such as strongly ionic systems, near-field MLP calculations can limit overall accuracy. Additional errors may result from uncertainty in the local ordering of structures to reproduce the atomic environments found in solid phases.

The atomic polar tensor (APT) neural network (Schienbein, 2023) can be a useful addition to an MD simulator and is used to model atomic polar tensors in liquid water. The APT is a charge distribution pattern that determines the distribution and orientation of molecules in molecular systems. Using this machine learning model, infrared (IR) spectra are obtained that are in good agreement with the reference calculations. APT reduces the IR spectrum to the motion of an atom (given by the atomic velocity) and the dipolar changes caused by that motion (given by the APT), corresponding to an IR selection rule. Consequently, this tensor is nothing more than a fundamental physical property that is strictly defined for any atom. Moreover, it does not depend on the distribution of molecules or charges, so it can quite well and accurately describe any atomic system.

Additionally, the development of high-dimensional neural network potentials (HDNNPs) in 2007 opened up the possibility of applying MLPs to the simulation of large systems containing about a thousand atoms [reviewed in Behler (2021)]. The first generation constitutes the earliest developments of neural network potentials intended for low-dimensional systems. Multidimensional potentials of neural networks gave rise to the second generation and worked according to the following principles: the total energy was expressed through the sum of atomic energy contributions depending on the environment; the description of atomic media was carried out through atom-centered symmetry functions satisfying rotational, translational, and permutation invariants; and the iterative construction of electronic structure data sets was carried out through active learning. Additionally, third-generation HDNNPs incorporate long-range interactions using environment-dependent partial charges expressed by atomic neural networks. Fourth-generation HDNNPs, which are just emerging, may already include global processes such as long-range charge transfer. However, one of the most demanding steps in the development of HDNNP is the creation of large reference datasets, where it is important to try to avoid noisy data that may interfere with the convergence of weights during optimization.

*Ab initio* molecular dynamics (AIMD) simulation is a rapidly evolving algorithm that represents one of the most important theoretical tools developed in recent decades. In the AIMD calculation, dynamic finite temperature trajectories are generated using forces found in electronic structures and instantaneously calculated with each subsequent simulation iteration. Thus, AIMD allows the identification and formation of chemical bond co-occurrences and takes into account the effects of electronic polarization (Grotendorst, 2000). AIMD has been successfully applied to a wide range of problems in physics and chemistry, and is now also in demand in biology. Numerous studies have revealed new physical phenomena and elucidated microscopic mechanisms that could not be discovered using empirical methods, often leading to new interpretations of experimental data and even suggesting new experiments.

AIMD simulation can predict the structure of a liquid by simulating a molecular system, solving the Schrödinger equations for each particle, and taking into account interactions between atoms, including hydrogen bonds, electrostatic, and van der Waals forces (Iftimie et al., 2005). AIMD can also predict the pattern of atomic movement over time, thereby providing information about the dynamics of a liquid system. This may offer an insight into diffusion or viscosity phenomena (Mazurek et al., 2021). The spectroscopic properties of a liquid can be obtained by calculating the vibrational frequencies and intensities of various modes (Raposo-Hernández et al., 2022), allowing simulated spectra to be compared with experimental data to verify the accuracy of the model.

However, like for any model of this nature, there are disadvantages associated with the accuracy of the electronic structure method and high computational costs. Perhaps the use of a higher-level electronic structure method instead of density functional theory (Choi et al., 2005) will reduce the calculation error and bring the results closer to reality.

## 3 Experimental approaches and results of their application

As is well known, a theory should be testable and verifiable through experimentation. However, “good rule not to put overmuch confidence in a theory until it has been confirmed by observation” (Sir Arthur Eddington, 1935). This consideration is especially relevant in the context of the study of complex systems such as those discussed in this review. Hypotheses come and go, stimulating the researcher’s thinking and directing towards new experiments and a fresh analysis of the available results. In the authors’ opinion, this section is of fundamental importance in terms of the reliability of individual experimentally established properties of water, on the one hand, and the insufficient accuracy of theoretical analysis of these properties within the framework of the proposed models, on the other hand. The main question is: what exactly can be measured using a certain experimental method, and what conclusions can be drawn from it? In our case, the set of methods is far from complete, and each method is closely associated with a specific task.

The traditional set of methods for studying the properties of solutions is currently being continuously modified and improved. The study of processes in the femtosecond range, which has been developed recently for a variety of experimental procedures, makes it possible to obtain information about fast exchange processes, generally providing extremely informative results that attract special attention. However, traditional methods of analysis are also being improved, as will be shown below, and their results are becoming more informative in terms of water structure analysis.

### 3.1 X-ray diffraction and neutron scattering in measuring the size distribution of water clusters

Despite some significant differences in the produced data, the methods with a resolution of the order of several angstroms, including X-ray diffraction, neutron scattering, small-angle scattering, emission spectroscopy, adsorption spectroscopy, and Raman spectroscopy, have common features associated with small correlation distance. Obviously, due to the absence of long-range order in liquids, the radial distribution function (or the Fourier-transformed image of this function, the so-called structure factor) is the main measured parameter. The pair correlation function implies very limited possibilities for describing the structure of monatomic substances. The resulting structure does not differ in any way from the structure of liquid argon (Banerjee and Bagchi, 2019). In general, the method allows obtaining an integral but instantaneous picture of the measured sample. Therefore, to obtain information about the structure of water, additional methods are required to describe a liquid consisting of polyatomic molecules. From the obtained experimental data, there are ways to identify three correlations: O-O, O-H, and H-H. Such methods do not provide angular correlations, but the experimental results are a good test of the model’s correctness (Wernet et al., 2004).

The main object for short-range order analysis in water at room temperature using the methods under consideration is the standard tetrahedral structure of water. This structure fluctuates, and its fluctuations increase with increasing temperature. Based on the “theory of two liquids,” standard for water research, there are attempts to classify this division differently: tetrahedral and distorted, located, favored and normal, high density and low density, taking into account changes in the number of molecules in each of these two classes when conditions and their fluctuations change (Leetmaa et al., 2008). There is no consensus on this issue, and some arguments can be provided by supporters of one or another theory. Issues of asymmetry have been discussed in recent years based on X-ray Raman and X-ray absorption spectroscopy (Wernet et al., 2004) as well as X-ray emission spectroscopy data (Tokushima et al., 2008), which demonstrate the presence of asymmetry and the existence of asymmetric clusters in different arrangements. A heated discussion in the literature (Leetmaa et al., 2008) regarding the interpretation of these results showed the ambiguity of the conclusions obtained from the analysis of the radial distribution function. However, Kühne and Khaliullin (2013) provided a point of view that helps reconcile the two existing and seemingly opposite models of liquid water: the traditional symmetric and the recently proposed asymmetric. The authors suggest that the asymmetry is a result of small instantaneous distortions of hydrogen bonds, which appear as fluctuations around the average symmetric structure. The use of small-angle X-ray emission and neutron scattering seems to be most convenient for studying cluster asymmetry (Huang et al., 2009). The above-mentioned manifestation of asymmetries will be used in the discussion of a hypothetical chiral hierarchical model of supramolecular water structures, proposed below in the Discussion section.

X-ray diffraction and neutron scattering hold a prominent place in studies of the hydration of impurity ions (Banerjee and Bagchi, 2019). Study of structural heterogeneity using X-ray scattering by electrons allows, according to the Bregg-Wulf condition, to determine the location of electrons in the cloud, thereby making it possible to estimate the sizes and locations of these structures. However, this does not allow studying molecular associates in fast dynamics, since it does not take into account the reorientation factor as a result, for example, of the action of an external electric field. These methods are much more informative for studying structure than the method of femtosecond spectroscopy, which will be discussed below.

From the point of view of the structure lifetime, the X-ray methods are of interest only in the part that is associated with finding the universal cluster size distribution, which should arise in equilibrium. This area of research is currently under development, and its main problems are: increasing the method sensitivity and taking into consideration the side effects that lead to distortion of the results. It should be mentioned that these methods are convenient for testing various models of MD (Clark et al., 2010). Various options for using these methods with short (femtosecond) pulses will be discussed in the Femtosecond spectroscopy section (see Section 3.6).

### 3.2 NMR

Nuclear magnetic resonance (NMR) is one of the most widely used structural methods. It was developed a long time ago, and its capabilities are currently well known. The nuclei must have a nonzero spin, and then the process of their relaxation after a pulse action can be observed in a magnetic field. In a number of studies, NMR was used to analyze the structural features of water and aqueous solutions. It is important to note that the structural features of water can be assessed using NMR only integrally. For example, it would be very difficult to assess processes that occur approximately 1,000 times faster (and the lifetime of a hydrogen bond is approximately a picosecond) using radio-spectroscopic methods with a frequency of approximately 500–1,000 MHz. However, it is possible to study the properties of water structure by assessing the relaxation times that are characteristic of water protons and strongly depend on the environment. For example, сhanges in relaxation processes and an increase in the content of nanoscale structures were observed after processing using high dilution technology (Iftimie et al., 2005). Also, it was found that, due to different masses and different interaction potentials, ions relax differently. Therefore, this method is applicable for studying various ions in water (Banerjee and Bagchi, 2019; Pluhařová et al., 2017; Stirnemann et al., 2010). It is much more interesting to discuss studies focusing on the process of molecular motion in aqueous solutions using NMR (Pluhařová et al., 2019). It appears that the temporal resolution of NMR may not be sufficient to answer the question on the ways of molecule reorientation.

In general, when studying water structures using NMR methods, one can conclude that water and water structures are far from thermodynamic equilibrium (Tiezzi et al., 2010). Proton relaxation in free water may differ from that in water that is part of supramolecular structures associated with other molecules or ions (Rodin, 2020). NMR studies of water structures indicate the existence of a long-range correlation, confirming the existence of many more water molecules in the structures than predicted by electrostatic theories (which conflicts with Debye theory) (Tiezzi et al., 2010).

There is a variation of the NMR technique, NMR relaxation of protons and nuclei of other elements (Bakhmutov, 2004), which has a great advantage in the accuracy of the analysis of aqueous solutions and the study of the state of ions and molecular structure in a liquid, in comparison with such well-known methods, as diffraction of X-rays, protons or electrons. Using this approach, the intensities and patterns of molecular motions are studied based on the dependencies of the relaxation rate of particles in a magnetic field on various parameters (temperature, concentration). The nature of diffusion and lifetimes in microstructural regions may indicate the mobility of particles. From the temperature dependencies obtained using NMR, it is possible to obtain information about the activation energy for the movement of water molecules in the hydration shells of alkaline earth metals (Buchner and Barthel, 2001). Thus, having obtained these physico-chemical parameters for aqueous solutions of salts and other compounds, it is possible to draw conclusions about the molecular movements of water in such complex structures.

### 3.3 Dielectric spectroscopy

**FIGURE 2:**
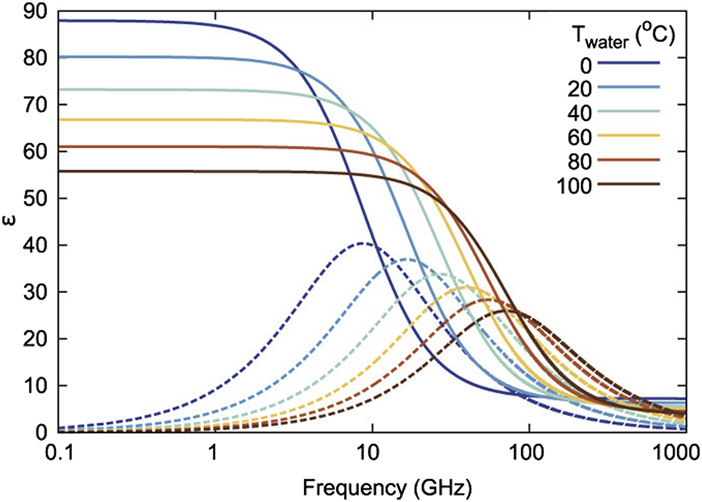
Water complex permittivity at various temperatures. The solid lines show the real part, and the dotted lines show the imaginary part (Andryieuski et al., 2015).

This term is applied to a variety of methods related to the study of the propagation and absorption of electromagnetic waves in different frequency ranges and the study of the dependencies of the complex dielectric permittivity on frequency (Buchner and Barthel, 2001; Kaatze, 2007). Such dependencies carry a considerable amount of information about the properties of the liquid. Any dielectric characteristics of a substance are associated with polarization, i.e., with a change in the dipole moment. Since water consists of molecules with a constant dipole moment that form multiple hydrogen bonds, dielectric spectroscopy is very sensitive to changes in the orientation of molecules, and the level of their interconnection, which are directly related to the structure of water. Generally, the frequency range of dielectric spectroscopy used to analyze aqueous solutions is between 10^6^ and 10^11^ Hz (El Khaled et al., 2016; Zelsmann, 1995; Ishihara et al., 1997; Behrends and Kaatze, 2005; Sato and Buchner, 2004; Lyashchenko and Lileev, 2010; Levy et al., 2012). Figure 2 shows the characteristic dielectric spectrum of water in the range of the main absorption band, which is commonly called the Debye relaxation band.

In the simplest way, this band is described by the Debye function:

$$\varepsilon \left(\omega \right)=\frac{\varepsilon_{0}-\varepsilon_{\infty }}{1-i\omega \tau }+\varepsilon_{\infty }$$ (1)

where ε_0_ is the static dielectric constant, ε_∞_ is the high-frequency dielectric constant describing the polarization of the substance due to all polarization processes faster than the Debye relaxation, τ is the Debye relaxation time, and ω is the cyclic frequency.

In some cases, instead of Equation 1, the generalized Havriliak-Negami Equation 2 (Fu et al., 2022) with two additional parameters 0 < (α, β) < 1 is used for a more accurate description of dielectric relaxation:

$$\varepsilon \left(\omega \right)=\varepsilon_{\infty }+\frac{\varepsilon_{s}-\varepsilon_{\infty }}{{\left(1-{\left(i\omega \tau \right)}^{\beta }\right)}^{\alpha }}$$ (2)

One of two special cases is often used: when α = 1, the relaxation time distribution is considered (the Cole-Cole equation), or when β = 1, the asymmetry of the spectrum is taken into account (Cole-Davidson equation). There are also more complex expressions to describe the frequency dependence of the dielectric permittivity of polar substances, but they are practically not used.

The Debye relaxation time of water, τ, means the waiting time for the activation energy sufficient to break hydrogen bonds, after which the molecule can change orientation (Von Hippel, 1988a; Von Hippel, 1988b). This leads to a change in the dipole moment and induces a dielectric response of water to an external field with characteristic frequencies at the maximum absorption of the Debye band v = 1/2πτ. The relaxation time can be described by Equation 3:

$$\tau =\tau_{0}*\exp\left(\frac{E}{kT}\right)$$ (3)

where *E* is the activation energy required to break the bonds of a water molecule, and *kT* is the characteristic thermal energy.

The Debye relaxation band is extremely sensitive to changes in the structure of water. Figure 2 shows a clear example of the spectral reflection of differences in the structure of water with temperature changes. A 1°C difference is enough to induce pronounced changes in the Debye band parameters. Besides temperature, various additives exert a significant effect on the structure of water. External molecules and ions dissolved in water cause changes in the structure of water around them, which is called the formation of hydration shells. Dielectric spectroscopy has been used for decades to study the structure of water and the manifestations of hydration effects in aqueous solutions (Behrends and Kaatze, 2005; Sato and Buchner, 2004; Khodadadi and Sokolov, 2017; Mashimo et al., 1989; Berntsen et al., 2011; Andryieuski et al., 2015).

**FIGURE 3:**
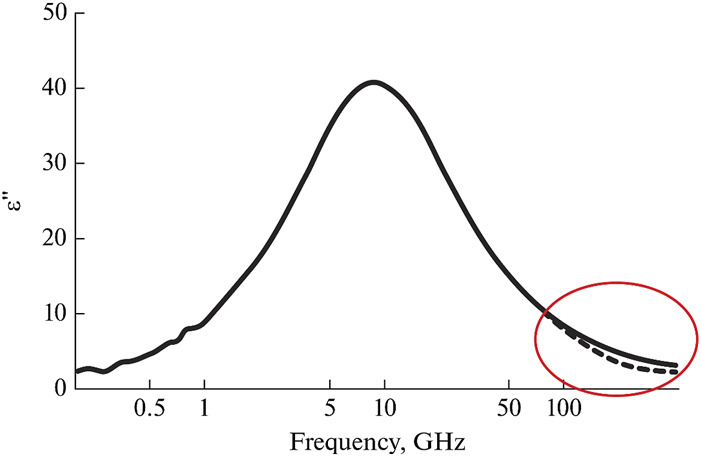
The imaginary part of the dielectric function of water at 0.2°С in the Debye relaxation band. High-frequency part shows the deviation of the dielectric function from the mono relaxation character. Adapted from (Buchner et al., 1999).

However, it has been found (Buchner et al., 1999), that when more broadband range (more than 100 GHz) is considered, the water spectrum cannot be well described using the Havriliak-Negami equation at any coefficients (Figure 3). Another type of relaxation with an absorption maximum at 0.5-1 THz is clearly present in the dielectric response of water (Buchner et al., 1999; Møller et al., 2009; Meissner and Wentz, 2004). The main hypothesis was the presence of a certain fraction of free or weakly bound water molecules, giving a dielectric response in the form of an additional band (Buchner et al., 1999; Yada et al., 2008; Penkov et al., 2015; Shiraga et al., 2015). Classical dielectric spectroscopy did not allow measurements of dielectric spectra to be carried out at frequencies above 0.5 THz with sufficient sensitivity, which significantly limited its capabilities. Other methods, such as IR and Raman spectroscopy, in turn, do not allow conducting measurements with high sensitivity at frequencies below 1 THz and, more importantly, do not determine the spectra of the complex dielectric permittivity. The development of THz spectroscopy methods was required to cover this “inconvenient” interval of about 1 THz.

### 3.4 THz spectroscopy

The terahertz range is a relatively narrow frequency range of 0.3-3 THz (10–100 cm^−1^) from a wide scale covered by modern spectroscopy of ∼10^−6^ – 10^20^ Hz. However, the THz range happened to be the most difficult for spectral analysis. From a technical point of view, neither electronic measurement methods applied from the low-frequency side nor optical methods applied from the high-frequency side of the THz range work well in the THz range. However, this method is highly effective at detecting collective vibrational modes in water, such as the translational and librational motions of molecules. These modes, occurring in the THz range, involve coordinated movements of multiple molecules and provide insights into the hydrogen bond network’s strength and dynamics by the different conditions (Zhao et al., 2020), moreover, this spectroscopy is sensitive to the picosecond hydrogen bond’s dynamics. However, THz spectroscopy has limitations: water’s strong absorption in the THz region can limit penetration depth, and scattering effects can also distort the spectra. These issues may complicate results interpreting.

In the early 2000s, powerful sources of THz radiation appeared, which allowed absorption spectra to be obtained in the THz range with sufficient sensitivity. This method proved to be very informative when analyzing the structure of water and, mainly, hydration shells. It showed that hydration shells are not limited to one or two layers of water with an altered state, but extend much further (Born et al., 2009; Conti Nibali and Havenith, 2014; Ebbinghaus et al., 2007; Heyden et al., 2010). Moreover, the larger the molecule, the greater the extent of its hydration shell. For biomacromolecules, it amounts to several nanometers (Heyden et al., 2012; Sushko et al., 2015; Penkov et al., 2021; Leitner et al., 2008). Furthermore, this does not mean a frozen “ice-like” hydration shell, but an area with altered intermolecular dynamics of water, which, certainly, is directly related to the hydration structure. In light of this, the volume fraction of hydration shells of macromolecules in solution is comparable or even greater than the volume fraction of the macromolecules themselves. This allowed taking a fresh look at the concept of heterogeneity of aqueous solutions, where water structure consists not only of water around dissolved molecules, but also of a separate phase of hydration.

Subsequently, a more advanced type of THz spectroscopy was developed: THz time-domain spectroscopy (THz-TDS) (Theuer et al., 2011; Lee, 2009). The method is based on the principle of coherent generation and detection of broadband THz radiation pulses using femtosecond lasers. The main advantage of THz-TDS over other THz methods is the ability to obtain not only absorption spectra, but also spectra of complex dielectric permittivity, which have much more informative value.

**FIGURE 4:**
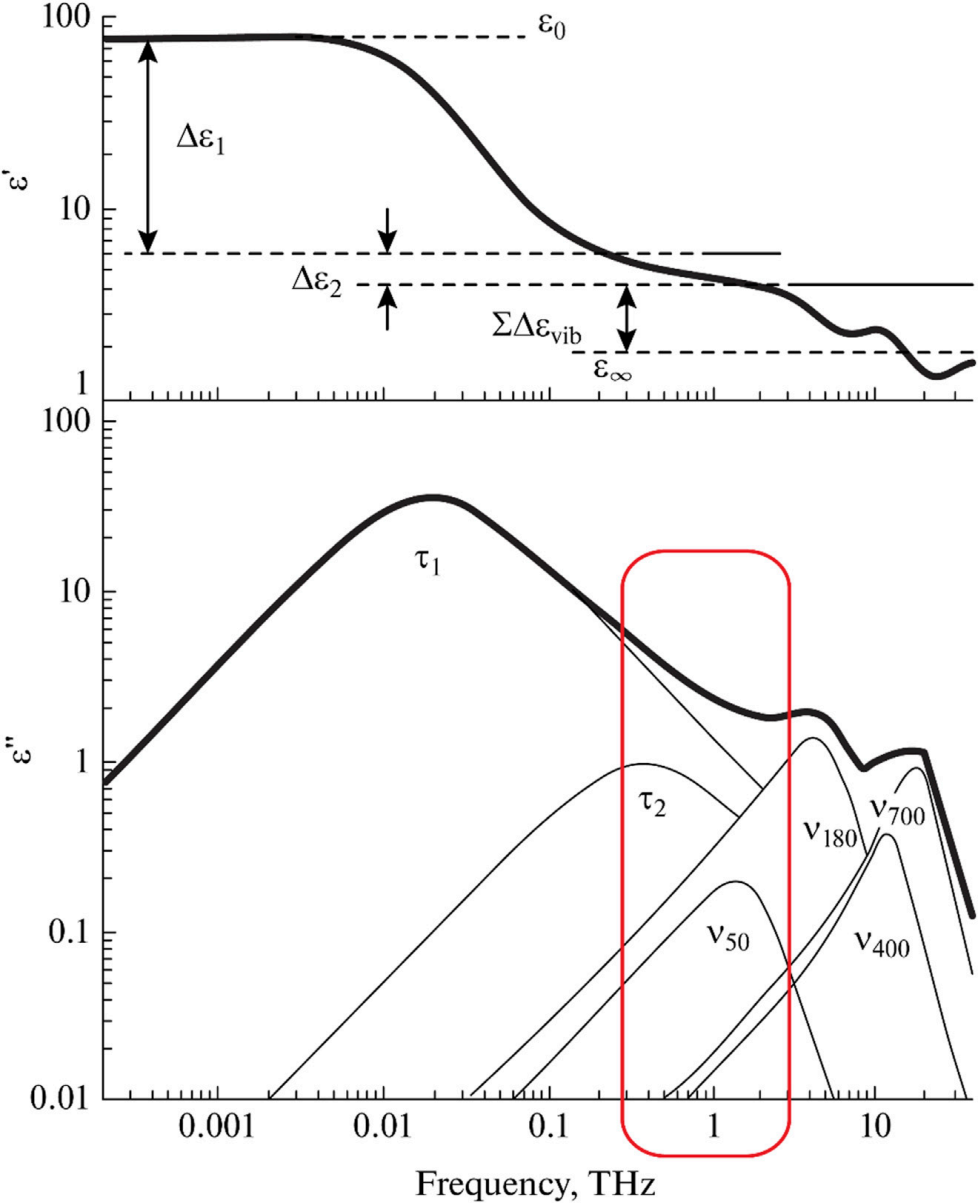
Decomposition of the dielectric spectrum of water into components. Adapted from (Fukasawa et al., 2005).

The dielectric response of water in the THz range consists of several overlapping bands (Figure 4). Now, each of them has general interpretations, although there are some alternative versions.

In addition to the Debye relaxation band discussed in Section 3.2, there is a higher frequency relaxation band at 0.5 THz. This band is usually associated with a certain fraction of free or weakly bound water molecules (Buchner et al., 1999; Yada et al., 2008; Penkov et al., 2015; Shiraga et al., 2015), although other hypotheses have been put forward (Zasetsky, 2011). The presence of water molecules with two different relaxation times can be considered a dielectric manifestation of the two structural composition of liquid water.

Using the parameters of the high-frequency relaxation band, the theory of calculating the number of free water molecules in the liquid phase was developed (Penkov et al., 2014; Penkov, 2022; Penkov and Penkova, 2023), and the number of free water molecules was calculated in various aqueous solutions (Penkov et al., 2021; Penkov, 2021; Penkova et al., 2021). The parameters of this band can be used to analyze the structure of water in solution and determine how it’s influenced by certain factors.

Vibrational bands with maxima at 50 and 180 cm^−1^ are attributed to the intermolecular vibrations of water molecules bound by hydrogen bonds. The first band refers to transverse vibrations, and the second band refers to longitudinal ones. Based on the parameters of the vibrational bands, one can draw conclusions about the energy and amount of hydrogen bonds in solutions and identify the effect of various additives on hydrogen bonds (Penkov, 2021; Penkova et al., 2021; Le Caër et al., 2011; Penkov et al., 2018; Penkov NV. and Penkova N., 2021; Penkov et al., 2022). The vibrational bands at 400 and 700 cm^−1^ (Zelsmann, 1995; Walrafen, 1967) reflect the librational vibrations of water molecules relative to two different axes and contain information about the averaged connectivity of water molecules with each other.

The spectral bands corresponding to all known types of intermolecular dynamics of liquid water are listed above. The remaining higher frequency bands within the IR range already belong to intramolecular vibrations. Surprisingly, all types of intermolecular dynamics are manifested precisely in the THz range. This explains why the THz range has an outstanding sensitivity to changes in the structure of water. As an illustrative example, the results obtained using various methods for determining the hydration number of a sucrose molecule can be provided: calorimetry – 6.3 (Zasetsky, 2011); viscometry – 11.2 (Penkov et al., 2014); acoustic methods – 13.8 (Penkov et al., 2014); THz spectroscopy – 35 (Penkov, 2022). Thus, THz spectroscopy is much more sensitive to changes in the structure of water than all other methods. The high sensitivity, together with the relatively recent development of this method, suggests great prospects for THz spectroscopy in terms of studying the structure of water.

To describe the dielectric response of water in the THz range, three main bands are usually considered (Equation 4): Debye relaxation, high-frequency relaxation, and the longitudinal band of intermolecular vibrations:

$$\varepsilon_{\mathrm{mod}}^{*}=\frac{\Delta \varepsilon }{1-i\omega \tau_{1}}+\frac{\Delta \varepsilon_{2}}{1-i\omega \tau_{2}}+\frac{A}{\omega_{0}^{2}-\omega^{2}-i\omega \gamma }+\varepsilon_{\infty }+i\frac{\sigma_{0}}{\varepsilon_{0}\omega }$$ (4)

where τ is the relaxation time for water molecules (τ_1_ for bound and τ_2_ for free water molecules); Δε_1,2_ is the contribution of these relaxation processes to the overall dielectric response (Δε_1_ for bound, and Δε_2_ for free water molecules); A is the amplitude of the vibrational band; ω is the resonant frequency; γ is bandwidth; ε_∞_ is the high-frequency dielectric permittivity (on the high-frequency side of the vibrational band); ω is a cyclic frequency; and i is an imaginary unit. In the case of the ionic conductivity of an aqueous solution, it is described by the last term, where σ_0_ is dc-conductivity, ε is an electrical constant.

**FIGURE 5:**
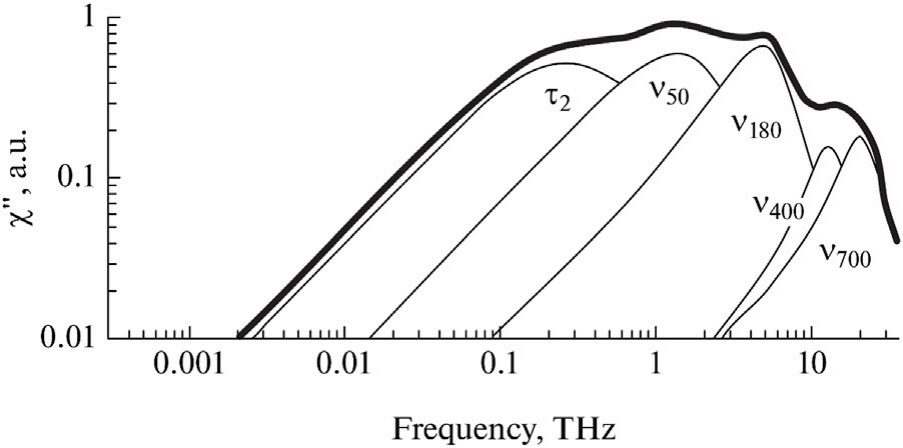
Decomposition of the Raman spectrum of water into components. Adapted from (Fukasawa et al., 2005).

The remaining bands make a minuscule contribution to the dielectric spectra in the THz range. Libration bands are more readily available for vibrational spectroscopy in the far IR range. In terms of calculation, taking them into account causes uncertainty in determining the parameters of the bands, including the three main ones. As a rule, they are not taken into account when decomposing dielectric spectra. The band of transverse intermolecular vibrations can be much better analyzed using Raman spectroscopy (Popov et al., 2016), since the relative contribution of this type of vibration to the change in molecule polarizability is much greater than the contribution to the change in the dipole moment. As can be seen in Figure 5, the contribution of a band at 50 cm^−1^ in the Raman spectra is comparable to high-frequency relaxation and longitudinal translation.

Therefore, the most informative data in the THz range can be obtained based on two types of spectra: dielectric and Raman. A comparative analysis of the results of dielectric spectroscopy and Raman spectroscopy for liquids without hydrogen bonds with the results for water elucidates the essence of these phenomena (Popov et al., 2016). This comparative analysis helps to detect shifts in vibrational frequencies due to changes in hydrogen bonding or solute concentration, and also investigate the microscopic heterogeneity in aqueous solutions. In liquids without hydrogen bonds, the difference is easily explained by rotational diffusion, whereas long hydrogen bonds play an important role in water, and their contribution to dielectric relaxation proves to be very significant.

### 3.5 Vibrational spectroscopy

In this section, we will briefly describe methods that determine the intensity and wavelength of radiation (for Raman spectroscopy) arising after interaction with the object under study. There are good reasons to describe the studies related to vibrational spectroscopy using ultrashort pulses separately. The use of short wavelengths to determine the pair correlation function was discussed in Section 3.1. The range of frequencies corresponding to the vibrations of molecules is the most informative for studying various structures. Oscillation studies are related to the IR range and have already been partially discussed in Section 3.3. The comparison of the vibrational spectra of H_2_O with D_2_O and HDO has great importance (Bakker and Skinner, 2010). Such comparison helps to relate spectral characteristics to structure and dynamics at the molecular level. The effectiveness of vibrational studies is due to the fact that O-H vibrations are very sensitive to the environment, which means changes in frequencies can be used to assess the interaction of water molecules with external forces from other molecules. Also, vibrational spectroscopic maps were developed to interpret complex IR and Raman spectra, which were then defined more accurately to describe the tension of O-H expansion in water (Lin et al., 2009; Kananenka and Skinner, 2018). The frequency of O-H vibrations changes with time. This frequency behavior is called spectral diffusion (Bakker and Skinner, 2010). Further, at lower temperatures, when supercooled water is obtained, vibrations undergo significant changes, emphasizing the transition from high-density to low-density liquid (near the liquid-liquid critical point) (Auer and Skinner, 2007). When ice forms in bulk water, the O-H bands become clearer, which emphasizes different organizational symmetries. Such water has been studied (modeled) quite well, but more complex high-density ices still pose a challenge for modeling. So it will be difficult to model interfaces or heterogeneities (Ni et al., 2018). In general, any calculation in such a complex system as water will always be only approximate in accuracy and requires additional improvement. Already today, machine learning techniques in predicting vibrational spectra are successfully used to improve accuracy (Baiz et al., 2021).

To obtain IR absorption spectra of aqueous solutions, different measurement configurations are used: classical absorption (Yuhnevich, 1973), ATR (attenuated total reflection) (Levina et al., 2018), and even radiation (Penkov NV. and Penkova NA., 2021; Penkov and Penkova, 2020a; Penkov and Penkova, 2020b). These spectra are spectral reflections of the same substance, but in different cases, it is more convenient to use one or another measuring configuration.

The IR spectrum of water at room temperature and a pressure of 1 atm reaches its maximum at the wavenumber of 3,400 cm^−1^ and has a slight convexity at 3,250 cm^−1^ with a half-width of 375 cm^–1^ (Bertie and Lan, 1996). The parallel polarized spectrum is bimodal, with sharp peaks around 3,400 and 3,250 cm^–1^, and half-widths around 425 cm^–1^, while the perpendicularly polarized radiation peak is located around 3,460 cm^–1^, and is rather asymmetric with a half-width of 300 cm^–1^. In the gas phase, the water molecule has completely different spectra, with peaks at 3,657 and 3,756 cm^–1^. The interpretation of these spectra is complicated and causes controversy (Bertie and Lan, 1996; Green et al., 1986; Hare and Sorensen, 1992; Gallot et al., 2002).

When interpreting IR spectroscopy data and attempting to sufficiently accurately solve a quantum-mechanical problem (using the Condon approximation), it is necessary to take into account quantum mechanical effects associated with the influence of nuclei. Therefore, the Condon approximation is reliable when the solvent influences the nuclear potential, but not when the electronic structure is significantly altered (Hamm and Zanni, 2011). Indeed, in order to understand what causes the special features of the spectrum (not the peaks, but its shape), numerical simulation and experiments with femtosecond effects have to be used.

### 3.6 Femtosecond spectroscopy

This term currently comprises a wide range of experimental methods with short to medium impact times and even faster response recording. In this case, the reaction of the system to a short external impact is relaxation. The characteristic times of such relaxation are also obtained in the analysis of continuous high-frequency signals. However, in this case, there is a very clear identification of the response signal. Let us sequentially discuss the application of femto- and attosecond impacts on water molecules (Bakker et al., 2008).

As was noted in the Introduction section, the results of femtosecond spectroscopy are considered the main proof of the rapid mode changes in water. When studying the interaction of water with hydrophobic compounds, the iceberg model was proposed (Frank and Evans, 1945). In this model, the hydrophobic part is compressed, water molecules are attached to the hydrophilic part, and the movement of the hydrophobic molecule occurs, like an iceberg, when bonds are broken. Diffraction methods did not show any difference in the structure of hydration shells from so-called free water, while NMR and measurements of dielectric relaxation showed the opposite (Ishihara et al., 1997; Kaatze et al., 1986; Haselmeier et al., 1995; Wachter et al., 2006). These methods indicated that the average mobility of molecules in the shells decreased. For example, attosecond spectroscopy in a comparative analysis of gaseous and liquid water showed a delay in the onset of photoemission by approximately 50–70 attoseconds (Jordan et al., 2020). Femtosecond spectroscopy made it possible to understand the main reasons for such a difference. The presence of biexponential decay composed of a fast component with a time constant 2.5 ps and a slow component with a time constant >10 ps is noted (Rezus and Bakker, 2007). The fast component of 2.5 ps has also been observed in the reorientation of pure water, indicating that this component is to be associated with the reorientation of the bulk water molecules in the solution (Rezus and Bakker, 2007; Rezus and Bakker, 2005) and the second time depended on concentration and exceeded 10 ps (Rezus and Bakker, 2007). According to some authors (Sciortino et al., 1991), fast time is associated with defects in the tetrahedral structure. According to this theory, the movement of water molecules occurs unevenly or intermittently (Skarmoutsos and Guardia, 2020; Hammerich and Buch, 2008).

In particular, this method can be used to study rearrangements of hydrogen bonds; for example, in the study by Fecko et al. (2003) it has been shown that changes in the local electric field lead to ultrafast reorganization of hydrogen bonds. For instance, a comprehensive review (Roberts et al., 2009) discussed how using 2D IR spectroscopy one could capture the time evolution of hydrogen-bonded structures and probe the microscopic interactions that govern the macroscopic properties of water. The work highlighted the complexity of water’s hydrogen-bond network, revealing that vibrational energy transfer and spectral diffusion are influenced by both local and collective molecular motions. Additionally, Asbury et al. (2004a) demonstrated the utility of ultrafast IR spectroscopy in capturing the interplay between different vibrational modes and their coupling with the surrounding environment, which has provided critical insights into the vibrational relaxation processes and the role of anharmonic couplings in water’s unique spectral characteristics. Vibrational echo spectroscopy using ultrashort pulses also makes it possible to obtain additional information about the properties of water. However, simulation data (Asbury et al., 2004b) are also used for interpretation, which suggests that the parameters are not completely independent, but the results of such measurements serve as a good test for theoretical calculations. In this case, the evolution of vibrations with a change in hydrogen bonds (the so-called frequency correlation function) is studied.

Since hydrogen bond jumping occurs on a picosecond timescale, femtosecond spectroscopy is a good tool for studying their dynamics. This technique uses ultrafast laser pulses to initiate and then probe the dynamics, allowing for the observation of transient states and processes in real-time (Stolow et al., 2004). There is no doubt that this area will develop rapidly. However, there are some key challenges in interpreting the results, including the complexity of the data, which involves distinguishing between various overlapping electronic and nuclear motions, and the requirement for precise control over experimental conditions to achieve high temporal and spatial resolution.

### 3.7 Light scattering methods

Spectral methods provide information about the structure of water, averaged by volume. In the presence of several structural phases, spectral methods can sometimes determine their relationship. However, they cannot determine the characteristics of heterogeneity. If, for example, one phase is distributed into another in the form of dimensional inclusions, then a complete understanding of the liquid structure is impossible without determining such parameters as the size, concentration, and contrast of one phase relative to another. Light scattering methods are used to determine these parameters.

Light scattering methods are actively used to study aqueous solutions. The presence of certain nanoassociates in practically pure water has been described (Ryzhkina et al., 2011; Konovalov et al., 2014). In aqueous solutions of salts, the phase of air nanobubbles, the so-called bubstons (Haselmeier et al., 1995), is spontaneously formed (Yurchenko et al., 2016; Bunkin et al., 2016a; Bunkin et al., 2021). Submicron heterogeneities can be found in most aqueous solutions of low molecular weight organics, which, for unknown reasons, maintain long-term stability and behave like Brownian particles (Sedlák, 2006; Subramanian et al., 2011; Bunkin et al., 2016b; Bunkin et al., 2018; Kononov, 2015; Penkov, 2023).

The most sensitive method for studying nanoscale objects in solutions is dynamic light scattering (DLS) (Carpenter, 1977). This method measures the correlation function of the intensity of scattered laser radiation in a solution containing the analyzed heterogeneities. Since heterogeneities are driven by Brownian motion, fluctuations in their concentration and, consequently, fluctuations in the refractive index occur. The radiation scattered at these fluctuations is recorded at a fixed angle, and the time correlation function of the intensity G(τ) is determined. Time correlations are completely determined by the diffusion coefficients of heterogeneities. For a monodisperse particle distribution, the correlation function G(τ) is related to the diffusion coefficient as follows from Equations 5, 6:

$$\sqrt{G\left(\tau \right)-b}=a\;e^{-q^{2}D\tau }$$ (5)

$$q=\frac{4\pi n}{\lambda_{0}}\sin\frac{\varphi }{2}$$ (6)

where τ is the delay time, a and b are experimental constants, q is the scattering vector, D is the particle diffusion coefficient, λ_0_ is the wavelength of laser radiation in vacuum, n is the refractive index of the liquid phase, and φ is the scattering angle.

Thus, the method allows determining the diffusion coefficient D of particles (stable heterogeneities in solution), and, more generally, the distribution of diffusion coefficients. Then, based on the Stokes-Einstein relation, the hydrodynamic diameter d_h_ (size distribution) is calculated:

dh=kT3πηD (7)

DLS, which is the most sensitive method, was used in almost all cases when the dimensional distributions of water heterogeneities should be determined (Bunkin et al., 2018; Konovalov and Ryzhkina, 2014; Postnikov et al., 2018). Moreover, by analyzing the changes in this distribution over time or under varying conditions, DLS can provide valuable insights into particle-particle interactions, such as aggregation, stability, and the formation of complexes. The technique is sensitive to changes in the solution environment, such as pH, ionic strength, and temperature, which can affect these interactions. For example, the detection of an increase in particle size could indicate aggregation (Kotoucek et al., 2021). However, it should be highlighted that the hydrodynamic diameter is one of the possible expressions of the size of an object. It may differ slightly from the size determined by other methods, and be both larger and smaller.

Another way to determine the size of optical heterogeneities is to calculate their average radius of inertia, Rg. To do this, one can use the dependence of the scattering intensity I on q (Equation 8) based on the approach known in scattering theory (Jin et al., 2007):

$$\lim_{q\to 0}I_{N}\left(q\right)=N\left(1-q^{2}\frac{1}{3}R_{g}^{2}\right)$$ (8)

The scattering intensity form factor appears in the theory, so the coefficient N is necessary for normalization per unit of the intensity measured in the experiment at q→0. This approach was used to determine the radius of inertia of density heterogeneities in various aqueous organic solutions (Sedlák, 2006; Subramanian et al., 2011; Penkov et al., 2024). A comparison of the hydrodynamic diameter and the geometric radius calculated from Rg allows us to draw conclusions about the structure of heterogeneities, for example, their level of looseness (Penkov et al., 2024; Chaikov et al., 2019) or fractality (Jin et al., 2007).

Another light scattering method actively used in the recent studies of heterogeneities in aqueous solutions (Li and Ogawa, 2000; Hagmeyer et al., 2012; Rak and Sedlák, 2023) is nanoparticle tracking analysis (NTA). This method analyzes the Brownian motion of particles using a microscope and determines the diffusion coefficients based on their mean square displacement. Then the size distribution is calculated based on the Stokes-Einstein relation, similar to Equation 7, but for the two-dimensional case. In addition to the sizes, NTA allows the concentration of particles to be determined due to their direct observation in a known volume of the sample. This is the main advantage of this approach, since it is almost impossible to determine the concentration using other light scattering methods. Although there are a few examples of concentration determination using DLS (Penkov et al., 2024), this requires much effort and is not always possible.

In addition to sizes and concentration, an equally important characteristic is the optical contrast of heterogeneities relative to the surrounding aqueous solution. This was determined using phase microscopy (Bunkin et al., 2016b). For more low-contrast heterogeneities in aqueous solutions, a combined method has recently been developed based on multi-angle static light scattering, DLS, and Mie theory (Penkov et al., 2024). This approach proved to be able to determine the optical contrast of heterogeneities in aqueous solutions at the level of differences in the refractive index of about 0.005 or less.

## 4 Discussion

A molecular picture of the mechanism of water reorientation is still lacking (Laage and Hynes, 2006). Often, rapid rearrangements of hydrogen bonds are considered evidence of the same rapid rearrangement of structures in water (Ivanitskii et al., 2014), although it is obvious that these processes are not identical. To correlate these values, it is necessary to use modeling, whose accuracy is low when analyzing large structures. One such model (Laage and Hynes, 2006) is based on the application to molecules of the theory of jumps in rotational diffusion, developed by Ivanov (Ivanov, 1964) for heavy Brownian particles. With such strong simplifications, the extended jump model allows one to find correlations with modeling results. Thus, in this case, we consider not the standard diffusion model of molecular motion but the jump mechanism of water reorientation. The search for experimental confirmation is associated with the analysis of vibrational spectroscopy data [reviewed by Perakis et al. (2016)]. It is noted that the reorientational correlation function of an ensemble of water molecules decays exponentially, and this fact may mean that the reorientation of water molecules is still diffusional.

One of the well-known works in the field of femtosecond spectroscopy (Omta et al., 2003) can be mentioned as another example. To study the effect of ions on the hydrogen-bond interactions in water, the orientational correlation time of bulk water molecules for several aqueous salt solutions (Mg(ClO_4_)_2_, NaClO_4_, and Na_2_SO_4_) was measured. It was concluded that the fast exchange rate of molecules in the second hydration shell of an ion indicated an insignificant effect of ions on the structure of hydrogen bonds in water. During independent discussions of this work, it was convincingly argued that the conclusion about such an insignificant influence can hardly be considered justified because instead of one water molecule, another molecule enters the hydration shell while the structure is preserved (Banerjee and Bagchi, 2019). Of course, the conclusion about the existence of a structure around an impurity ion for an infinitely long time cannot be extended to structures in pure water, but it is obvious that the time of hydrogen bond jumping is not the time of the existence of structures in water.

Thus, it is impossible to draw a reliable conclusion about the lifetime of certain supramolecular structures in water from a review of experimental data. The time of hydrogen bond jumping is not directly related to the rate of molecular exchange, and the fact of molecular exchange does not yet indicate that the structure disappears. Small energy changes in large structures greatly complicate their experimental analysis.

The high frequency of molecular exchanges does not at all contradict the possibility of the existence of relatively long-lived supramolecular structures. In inanimate and live nature, as well as in engineered structures, a counterintuitive rule is often enacted: a dynamic system composed of elements that are separately unstable can be stable. This also applies to long-lived complex organisms composed of short-lived cells and molecules.

One should also be aware of the difference between hypothetical pure water and water with low and ultra-low concentrations of impurities. Further development in this direction is difficult due to the complexity of experimental measurements, but the effect of ultra-small impurities is rather obvious, as they are the centers of structure formation, and large Debye radii at low ion concentrations make it possible for them to actively influence physico-chemical and biological processes, including through their effect on highly hydrated molecules.

Theoretical models are often used to describe certain properties of water that are quite coarse in terms of bond energy (compared to the energy of macrostructures), and they are used at the expense of accuracy in describing other properties in all cases. A general analysis of models for the entire range of thermodynamic and electrical properties, transfer coefficients, and other parameters always shows a lack of versatility. In some cases, the introduction of two groups of molecules, both in terms of relaxation time and activation energies, is an effective method of description.

As mentioned above, the application of empirical force fields has influenced the study of the nuances of hydrogen bonds and their role in chemical and biological processes, and has made a great contribution to the understanding of the structural heterogeneity of water (Henchman, 2016) and modeling of anomalous water properties (Moilanen et al., 2008).

However, the issues of modeling accuracy do not lose their relevance, and research continues to improve the approaches to modeling. One such example will be the use of the RexPON force field based on quantum mechanics (Naserifar and Goddard, 2018). This model shows better accuracy, but also requires further development. For example, the value of the self-diffusion coefficient (D_s) calculated in the RexPON model turned out to be approximately twice as high as the experimental value, namely computed Ds = 4.21 × 10^−5^ cm^2^ s^−1^ is higher than the experimental 2.27 × 10^−5^ cm^2^ s^−1^. Or, overestimation of viscosity leads to a calculated restructuring, for example in models BLYP-D3 and SCAN (Zhang et al., 2021). Thus, the complexity of water systems still does not allow us to estimate the parameters of local water structures and their dynamics with sufficient accuracy, although consideration of nuclear quantum effects can significantly improve the accuracy (Li et al., 2022).

As we have already noted, more accurate models with a small number of molecules have limited capabilities for interpreting the influence of detected effects on dynamic and static parameters. In addition, it is obvious that they cannot describe large structures.

This situation demonstrates the limited application of classical ideas about the distribution of structures by energies and lifetimes, which is quite natural under the conditions of a complex system of connections. All this complicates the possibility of predicting the time of existence of certain structures in water, and even the issue of the equilibrium distribution of structures in water has not yet been resolved. Under these circumstances, one should exercise caution when considering the estimates of structure lifetimes provided by one model or another.

The answer to the question of the existence or absence of long-lived structures can only be given if an extraordinary but plausible and convincing hypothesis appears, followed by a reliable theory supported by experimental data and describing the unusual thermodynamic properties of water. Recently, the abundance of new models that are similar in many respects and the announcement of upcoming models as universal and consistent have generated a certain skepticism in the scientific community.

In this situation, when such a complex problem has no comprehensive explanation, innovative hypotheses are needed to provide unconventional approaches for conducting experiments or to inspire the researchers to rethink existing ideas. According to the existing models, supramolecular structures of water have homogeneous, monostructural, and single-level organization. It is possible to imagine a hierarchically organized regular structure of water, where different scale levels of supramolecular formations can form a hierarchy and have structures that differ by their symmetries.

The authors of this review put forward a general hypothesis, introducing a new perspective on the problem. It is based on the symmetry factor as well as the possible and probable interconnection of energy transformations and a series of symmetry breaks in the process of spatiotemporal self-organization during the formation of large hierarchically organized water structures, nanoassociates, or clusters, in particular involving the phenomenon of chiral dualism. The addition of symmetry properties is not only expedient for describing states of water but, just like energy, represents a structure-forming factor. This structuring factor will certainly depend on external physical conditions, such as temperature, mechanical impacts, or magnetic fields, which will always interact with water dipoles. However, hierarchical structure formation is an interconnected sequence of symmetry breaks and energy transitions, including a change in the form of energy within a system, contrary to the stationary idea of water memory. A complex system is capable of overcoming (bypassing) the kinetic barrier to transition from a classical thermal or concentration fluctuation to a giant fluctuation, acquiring a new structure with minimal energy costs. The structure can be dynamic, but stable under the conditions of local thermodynamic nonequilibrium.

As a science of living systems, biology unexpectedly made it possible for physics/biophysics to become aware of the crucial role of the phenomenon of chirality in the construction of discretely organized, stable, and long-lived hierarchical molecular structures regularly observed in biomacromolecules, as well as in chiral polymers and artificial liquid crystals (Tverdislov and Malyshko, 2020). Manifestations of the alternation of left-handed and right-handed intra- and supramolecular structures in proteins, nucleic acids, and polymer structures that are homochiral at the primary level directly suggest the significant role of water molecules in the formation of these chiral structures. In this context, instead of just filling their internal spaces and shells (even with specific electrical/electronic properties), water quite possibly becomes a fundamental factor of symmetry.

This approach is based on the model of quasicrystalline chiral water structures proposed several years ago by Lobyshev et al. (2003), Bulienkov and Zheligovskaya (2017) and the model of a typical construction of hierarchically organized chiral structures in proteins and nucleic acids (Tverdislov and Malyshko, 2020). In the first case, the authors considered the possibility of the formation of chiral supramolecular quasi-crystalline structures by slightly deformed water molecules that enable the formation of structures more complex than hexagonal. In the second case, a universal rule was educed and substantiated, according to which initially homochiral monomers can spontaneously form hierarchies of structures on different scales with alternating signs of chirality in the process of polymeric or liquid crystal structure formation, and this hierarchy acquires structural, thermodynamically supported stability.

This observation may be extended to water structures, so that the local water structure, instead of representing a homogeneous fluctuation, would be similar to a giant fluctuation acquiring a more complex and hierarchical structure. The proposed approach seems quite legitimate due to the fact that the dynamic structures of water are apparently represented by non-equilibrium local formations. We assume that our model is related to liquid, polymer, and liquid crystal chiral systems.

We would like to provide an example of the high stability and durability of such hierarchically organized chiral structures. The helices and coiled coils of protein molecules are individually unstable in water, but the protein molecule, which contains right-handed α-helices and left-handed coiled coils, is extraordinarily stable and long-lived, just as water molecules are. It seems reasonable to consider such an assumption as a working hypothesis regarding a stable, long-lived water macrostructure. Optical polarization methods may be one of the ways to experimentally test this hypothesis. Additionally, an independent analysis of the available experimental data from the perspective of the proposed hypothesis is warranted. In Section 3.1 we discussed circumstantial evidence in favor of this hypothesis.

**FIGURE 6:**
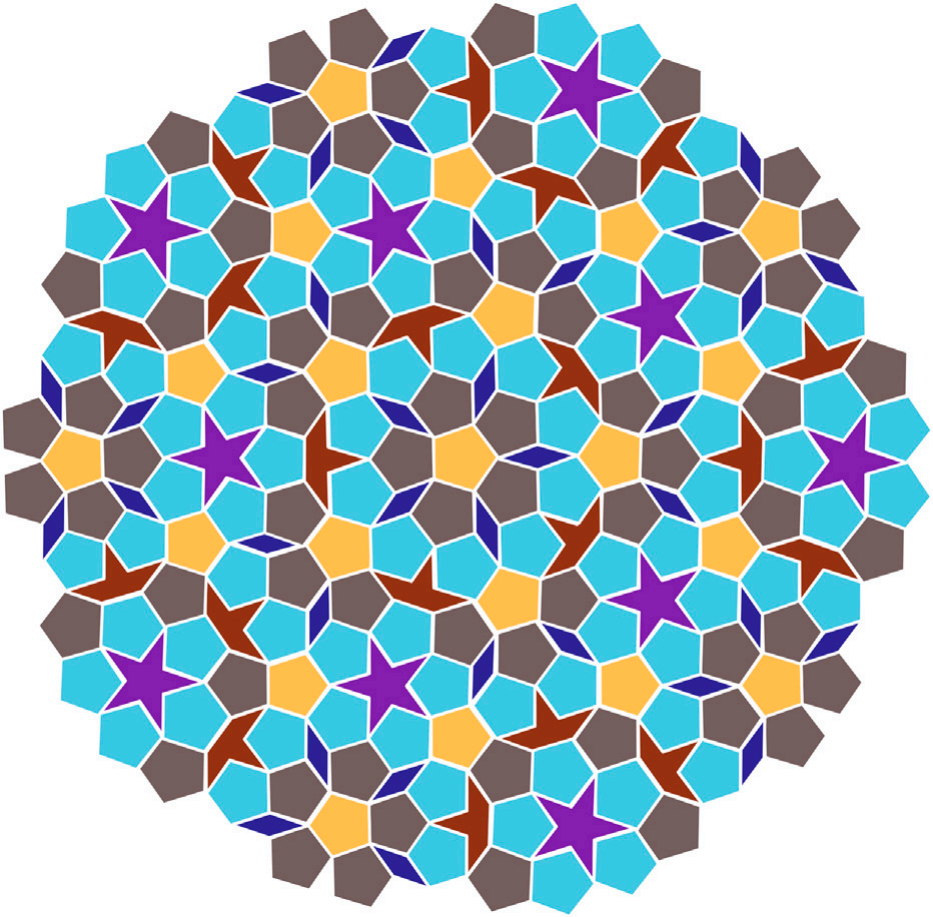
Mosaic of fifth-order symmetry elements (Lobyshev and Solovey, 2023).

**FIGURE 7:**
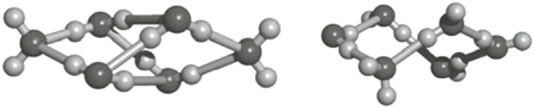
Hexacyclic water structures. **(A)** cage of water molecules. **(B)** twist bath of water molecules. Adopted from (Lobyshev and Solovey, 2023).

The discussed symmetric-chiral model, in principle, does not contradict the previous hypotheses of the formation of water aggregates and clusters, but develops them and, for the first time, proposes a very specific mechanism for the formation of hierarchically organized chirally alternating long-lived macroscopic structures (Figure 6) (Lobyshev and Solovey, 2023; Goncharuk et al., 2017). Like numerous previous models, the present model assumes the spontaneous formation of a macroscopic colloidal particle from water molecules with a certain structure of hydrogen bonds between water molecules (Figure 7). In it, water molecules form chiral rather than traditional hexagonal or amorphous structures. Known models, even those implying the emergence of symmetric structures, have never made symmetry and symmetry breaking a fundamental factor in the concepts of water structures (even if they somehow allowed for their presence in clusters, clathrates, flickering associates, emulons, etc.).

It was shown that water molecules, due to the relative flexibility of intermolecular hydrogen bonds, are capable of forming hydrogen-bonded clusters of increasing complexity, called modules. The modular approach assumes consideration of the complete connectivity of the elements of the module structure rather than the minimum energy of the system (Lobyshev and Solovey, 2023; Bulienkov, 1991; Bulienkov, 1998). A chiral hexacycle with a twist-boat conformation can be formed from dimers of water molecules with a torsion angle of about 38°. Such structures cannot continuously fill space, therefore they form locally stable non-crystalline structures (Figure 7). The minimal topologically stable structure is a system of 10 particles that are linked into five hexacycles. Successive complication of structures consisting only of such twist-boats leads to the formation of a 30/11 helical spiral as well as more complex modules from a larger number of particles. The spirals can be both right-handed and left-handed. Various non-crystallographic parametric structures can be created from modules. Helical in nature, they can form planar or spherical quasiparticles at higher levels of organization. In this case, such blocks are likely to be homochiral (although of arbitrary chirality) and have a relatively hydrophobic surface since the hydrogen bonds will be “drowned” inside the specified structures. A similar justification is developed in the work (Andersson, 2024), although it is not about chiral but about achiral solutions and aqueous structures. In turn, due to their hydrophobicity, these quasiparticles will tend to form clusters. It is possible that these clusters will have a fractal hierarchical structure. And it is the chiral conjugacy of discrete levels of cluster organization separated by kinetic barriers that can determine the stability of the entire system within the framework of our model.

The initial alternating chiral water structures of this kind could have been blocks of hydration shells of chiral biomacromolecules such as proteins, nucleic acids or phospholipids (Lobyshev and Solovey, 2023), which acquired individual independence during multiple nonequilibrium dilution of solutions.

At present, it is not possible to propose a direct experimental method for confirming the discussed structural symmetry hypothesis. It can be assumed that with the development of the theoretical model, quantitative thermodynamic and kinetic estimates will be obtained, with the help of which it will be possible to adequately interpret the data of optical polarization, NMR, and dielectric studies in a wide frequency range. Inclusion in the discussion of the model of information regarding the results of physical effects (heating, cooling, pressure, and multi-frequency electromagnetic effects) will presumably contribute to its validation.

Of particular interest are new experimental data devoted to the dynamic properties of water and aqueous solutions. In works (Epstein, 2023; Petrova et al., 2024; Shapovalov, 2023), the possibility of using vibration treatment to form clusters in aqueous solutions was considered. After vibration treatment, water samples (named iterations) acquired unique physico-chemical properties, including the ability to emit in the IR, THz, and microwave ranges. In addition, it became possible to classify iterations into several groups (fractions) based on differences in physico-chemical and biological properties (Petrova et al., 2024). The steady state of water after vibration treatment, as well as the ability to emit in the electromagnetic range, may also be related to its structural features. External rhythmic influences can transform the system from one nonequilibrium stable state to another, more harmonious holographic state. This suggests that the symmetry of the system can change in response to such influences, moving to a more ordered state (Epstein, 2023). These structural features have yet to be studied using modeling, as well as experimental work in the field of physics and biophysics.

## 5 Future directions

The study of hydrogen bonds is key to understanding the cluster structure of water. Indeed, as noted earlier, these bonds are very unstable and instantly break down, but the water molecules themselves continuously change their orientation, thereby forming new bonds. Therefore, improving the methods of molecular modeling and experimental methods for recording the restructuring of hydrogen bonds will make it possible to verify the ambiguous nature of liquid water, consisting of continuously emerging molecular clusters.

At the moment, there are no such high-precision methods of computer modeling and experimental testing that would allow theoretical approaches to be brought closer to experimental observations. We can assume that the introduction of AI will help in the future to systematize existing modeling methods, compose a synergistic algorithm, and create a generally accepted multi-level model, thereby increasing the accuracy of describing the dynamics of molecular heterogeneities in water. Moreover, this multi-level model based on an AI algorithm in combination with machine learning will provide a dynamic increase in the accuracy of simulations of intermolecular interactions in water, improving itself with each subsequent cycle of use.

## 6 Concluding remarks

Research on the structure and properties of water is actively ongoing. However, an accurate description of even the individual components of macrostructures has not yet been performed, which means that it is too early to address the accuracy of the calculations of the macrostructures themselves. Experimental methods give an increasingly accurate picture of molecular motions, but the frequency of jumping is not generally related to the lifetime of structures. This is especially evident in the analysis of ion hydration shells, which retain their structure for an infinitely long time despite the rapid jumping of individual water molecules. The differences in water modeling approaches result in trade-offs between accuracy, computational cost, and applicability. Classical models are widely used for their efficiency but may lack detailed accuracy. Quantum mechanical models provide detailed insights but are computationally expensive. Machine learning models offer a promising middle ground, with potential for high accuracy and wide applicability. The choice of model depends on the specific scientific or engineering context and the level of detail required for the study.

The main problem is that macrostructures have very low energies. The effects associated with their appearance are small, in terms of both energy and entropy. Existing methods make it possible to describe only relatively small structures with high binding energies, and even then, the description would be approximate. Under these conditions, the issue of describing macrostructures using the available methods is rather misleading. The second issue concerns the jumping of hydrogen bonds that takes place in picoseconds, but these times are not directly related to the movement of molecules. Moreover, if some molecules do jump, then others take their place, as happens in the hydration shells of ions. The same situation can occur in any structure, including chiral. So far, there are no experimental methods for direct measurement of their lifetime. For this reason, it is difficult, but necessary to discuss adequate methods for studying macrostructures.

Thus, despite the abundance of experimental and theoretical data, it is currently difficult to draw a definite conclusion about the presence or absence of long-lived structures in water at ambient temperature. The discussions about various types of femtosecond spectroscopy and modeling of hydrogen bond jumping do not offer any reliable conclusions in favor of a certain point of view on long-lived structures.
