# Supplementary material for: The heterogeneity of aqueous solutions: the current situation in the context of experiment and theory
Source: Front Chem. 2024 Sep 26;12:1456533. doi: 10.3389/fchem.2024.1456533 (PMC11464478; doi:10.3389/fchem.2024.1456533)
Supplement: Supplementary file 1 [file Table1.docx]

Supplementary Material

# Supplementary Figures and Tables

## Supplementary Tables

**Supplementary Table 1.** Consistency of the results of the simplest models using polarization potentials with the available experimental data, adapted from [3]. The first four columns show the characteristics of water that can be described by a model (if such a comparison is included, columns are marked with +). The correspondence to thermodynamic parameters is shown in the following 9 columns, where ρ is the density of the liquid, ΔH_vap_ is the enthalpy of vaporization, Cp is the heat capacity, D is the diffusion coefficient, ε is the dielectric constant, η is the viscosity, κ is the isothermal compressibility, α = coefficient of thermal expansion, and TMD is the temperature of maximum density. Green circles mean that accuracy is higher than 5 percent, blue circles stand for 5 to 10 percent accuracy, and red circles show a discrepancy of more than 10 percent.

| Model | Structure and Energetics | | | |  | Thermodynamic Properties (at 298 K) | | | | | | | | |
| --- | --- | --- | --- | --- | --- | --- | --- | --- | --- | --- | --- | --- | --- | --- |
|  | g(r) | dimer | clusters | ice |  | ρ | ΔH_vap_ | C_p_ | *D* | ε | η | *κ* | α | TMD |
| BK3 | + | + | + | + |  | • | • | • | • | • | • | • | • | • |
| AMOEBA | + | + | + | + |  | • | • | • | • | • | • | • | • | • |
| iAMOEBA | + | + | + |  |  | • | • | • | • | • | • | • | • | • |
| COS | + | + |  | + |  | • | • |  | • | • |  | • | • | • |
| AMOEBA14 | + | + | + |  |  | • | • | • | • | • |  | • |  | • |
| X-POL |  | + | + |  |  | • | • | • | • | • |  | • | • |  |
| T4NN | + | + |  |  |  | • | • | • |  |  |  | • | • |  |
| SWM4-DP |  | + | + |  |  | • | • |  | • | • |  |  |  |  |
| SWM6 |  | + | + |  |  | • | • |  | • | • | • |  |  | • |
| AMOEBA/GEM-DM | + |  |  |  |  | • | • | • | • |  |  |  |  | • |
| QCT | + | + | + |  |  | • |  | • | • |  |  | • | • | • |
| TTM3-F | + | + | + | + |  | • | • |  | • | • |  |  |  |  |
| TTM4-F | + | + | + | + |  | • | • |  | • | • |  |  |  |  |
| SWM4-NDP | + |  | + |  |  | • | • |  | • | • | • |  |  | • |
| POL5 | + | + | + |  |  | • | • |  | • | • |  |  |  | • |
| POLIR | + | + | + | + |  | • |  |  | • | • |  |  |  |  |
| POLI2VS | + |  |  |  |  | • | • |  | • |  |  |  |  |  |
| Pol3 | + | + |  |  |  | • | • |  | • |  |  |  |  |  |
| SCP-POL |  | + | + |  |  | • |  |  |  | • |  |  |  |  |
| TIP4P-POL |  | + | + |  |  | • |  |  |  | • |  |  |  |  |
| GEM* |  | + | + |  |  |  | • |  |  |  |  |  |  |  |
| TTM2 | + | + | + | + |  | • |  |  |  |  |  |  |  |  |
| MCY | + |  |  |  |  |  |  |  |  | • |  |  |  |  |
| MCYna |  |  |  |  |  |  |  |  |  | • |  |  |  |  |
|  |  |  |  |  |  |  |  |  |  |  |  |  |  |  |
| SCME | + | + | + | + |  |  | | | | | | | | |
| Polarizable Electropole | + | + | + |  |  |  |  |  |  |  |  |  |  |  |
| DDP2 | + | + | + |  |  |  |  |  |  |  |  |  |  |  |
| Yoon |  | + | + | + |  |  |  |  |  |  |  |  |  |  |
| EFP | + | + | + |  |  |  |  |  |  |  |  |  |  |  |
| Lybrand & Kollman |  | + |  | + |  |  |  |  |  |  |  |  |  |  |
| NEMO | + | + |  |  |  |  |  |  |  |  |  |  |  |  |
| SIBFA |  | + | + |  |  |  |  |  |  |  |  |  |  |  |
| OSS |  | + | + |  |  |  |  |  |  |  |  |  |  |  |
| VRT(ASP-W)III |  | + | + |  |  |  |  |  |  |  |  |  |  |  |
| Stillinger & David |  | + |  |  |  |  |  |  |  |  |  |  |  |  |
| Campbell & Mezey |  | + |  |  |  |  |  |  |  |  |  |  |  |  |
| Singh-Kollman |  | + |  |  |  |  |  |  |  |  |  |  |  |  |
| ASP-W2 |  | + |  |  |  |  |  |  |  |  |  |  |  |  |
| ASP-W4 |  | + |  |  |  |  |  |  |  |  |  |  |  |  |
